# Impact of ageing on homologous and human-coronavirus-reactive antibodies after SARS-CoV-2 vaccination or infection

**DOI:** 10.1038/s41541-024-00817-z

**Published:** 2024-02-20

**Authors:** Fan Zhou, Juha Vahokoski, Bergen COVID-19 Research Group, Siri Øyen, Marianne Sævik, Hanne Søyland, Helene H. Sandnes, Anders Madsen, Karl A. Brokstad, Kristin G. I. Mohn, Camilla Tøndel, Nina Langeland, Rebecca J. Cox

**Affiliations:** 1 Influenza Centre, Department of Clinical Science, University of Bergen, Bergen, Norway; 2 Department of Clinical Science, University of Bergen, Bergen, Norway; 3 Department of Medicine, Haukeland University Hospitalen, Bergen, Norway; 4 Department of Microbiology, Haukeland University Hospital, Bergen, Norway; 5 Eidsvåg Family Practice, Bergen, Norway; 6 Department of Medicine, Haukeland University Hospital, Bergen, Norway; 7 Department of Safety, Chemistry and Biomedical Laboratory Sciences, Western Norway University of Applied Sciences, Bergen, Norway; 8 Department of Paediatrics, Haukeland University Hospital, Bergen, Norway

**Keywords:** Humoral immunity, RNA vaccines, Viral infection

## Abstract

The endemic human coronaviruses (HCoVs) circulate worldwide yet remain understudied and unmitigated. The observation of elevated levels of HCoV reactive antibodies in COVID-19 patients highlights the urgent necessity of better understanding of HCoV specific immunity. Here, we characterized in-depth the de novo SARS-CoV-2 specific antibody responses and the boosting of HCoV-reactive antibodies after SARS-CoV-2 vaccination or infection in individuals up to 98 years old. All the vaccinees were home-dwelling with no documented SARS-CoV-2 infection before receiving the COVID-19 mRNA vaccine (BNT162b2). The first two vaccine doses elicited potent SARS-CoV-2 spike binding antibodies in individuals up to 80 years. The third dose largely boosted the previously low S2 domain binding and neutralizing antibodies in elderly 80–90 years old, but less so in those above 90 years. The endemic *betacoronavirus* (HKU1 and OC43) reactive antibodies were boosted in all vaccinees, although to a lesser extent in those above 80 years old. COVID-19 patients had potent elevation of *alpha-* and *betacoronavirus* (229E, NL63, HKU1 and OC43) reactive antibodies. In both patients and vaccinees, S2 domain specific antibody increases correlated with SARS-CoV-2 neutralizing and HCoV-reactive antibody responses in all ages, indicating S2 domain as a candidate for future universal coronavirus vaccine design.

## Introduction

The endemic human coronaviruses (HCoVs) circulate seasonally and generally cause mild common cold like symptoms. Seroprevalence studies suggest most people have encountered one or more HCoVs in childhood by the age of 5^1–3^. Several studies have reported high levels of HCoV reactive antibodies in critically ill or deceased Coronavirus disease 2019 (COVID-19) patients, suggesting a detrimental effect of HCoV cross-reactive antibodies on clinical outcomes^4,5^. Others have found the elevated levels of HCoV antibodies correlated with favourable outcomes in mild to moderate COVID-19 patients^6,7^. Furthermore, COVID-19 vaccine studies demonstrated less potent increases of HCoV antibodies after two vaccinations than natural infection^8,9^.

The surface spike protein of coronaviruses is the major target of immune responses. The spike S1 domain consists of N-terminal domain and receptor binding domain, which are immunodominant and contain multiple neutralizing epitopes. In contrast, the spike S2 domain is more conserved among *betacoronavirus* including HCoVs, severe acute respiratory coronavirus 2 (SARS-CoV-2) the ancestral Wuhan vaccine strain and variants of concern (VOC) as well as SARS-CoV-1 and Middle East respiratory syndrome coronavirus (MERS-CoV)^10,11^.

The elderly have higher risk of complicated COVID-19, including prolonged disease course, more critical illness and increased mortality, although it is often difficult to dissect impaired immunity from the effect of comorbidities^12–14^. Here, we characterized in-depth the binding and neutralizing antibody responses in adults up to 98 years old against SARS-CoV-2 and the endemic HCoVs *alphacoronavirus* (229E and NL63) and *betacoronavirus* (HKU1 and OC43). Our results highlighted the heterogeneity of kinetics, magnitude and breadth of de novo SARS-CoV-2 specific versus boosting of pre-existing HCoV antibodies in different age groups, after booster vaccination and natural infection.

## Results

### Study design

**Fig. 1:**
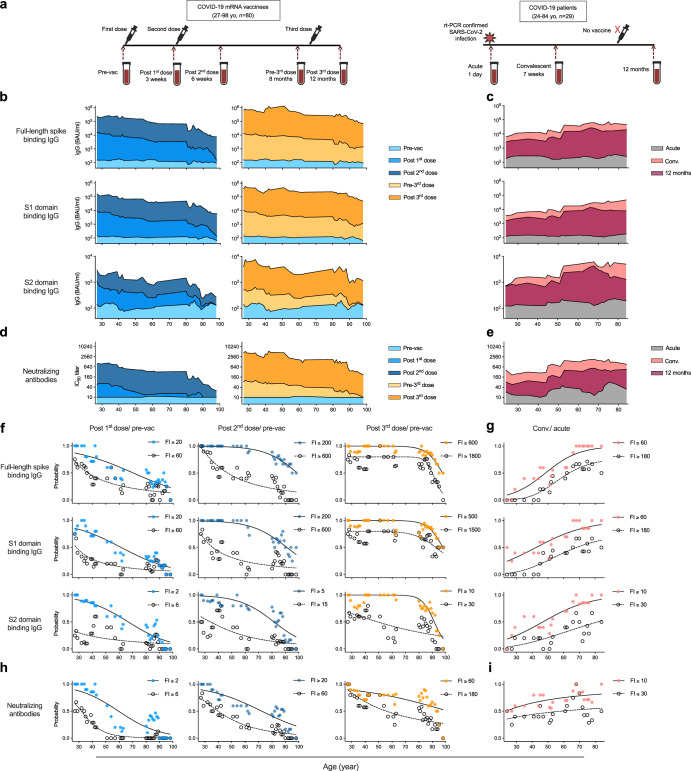
SARS-CoV-2 specific antibody responses vary across ages. **a** Illustration of the study design. **b**, **c** SARS-CoV-2 spike binding IgG concentrations in the vaccinees (**b**) and patients (**c**) were measured against the full-length spike (top), S1 (middle) and S2 domain (bottom) from the ancestral Wuhan-Hu-1 strain of SARS-CoV-2. **d**, **e** Neutralizing antibody titers in the vaccinees (**d**) and patients (**e**) were measured against the live SARS-CoV-2 Bergen-1 virus. **f**–**i** Probability of subjects having indicated fold-induction (FI) in spike binding IgG (**f**, **g**) and neutralizing antibodies (**h**, **i**). From all vaccinees (**f** and **h**) fold-inductions were calculated after the first (left), second (center), and third dose vaccines (right). From the infected patients (**g** and **i**) after the convalescent (conv.) phase. The antibody concentrations (binding antibody unit (BAU)/ml) and titers were calculated as geomean of the nearest 5 ages in (**b**, **e**). The probabilities were calculated for the group of subjects at the nearest 5 ages in (**f**–**i**). Nonlinear fitting curves are plotted. Duplicates were performed in all experiments.

To investigate the antibody responses against SARS-CoV-2 and HCoVs, COVID-19 vaccinees^15^ and SARS-CoV-2 infected patients^16^ were included in this study. Eighty vaccinees (age range 27 to 98 years old, 60% female) received three doses of monovalent COVID-19 mRNA vaccine (BNT162b2). Sequential blood samples were collected pre-vaccination, 3 weeks post 1st dose, 3 weeks post 2nd dose (6 weeks post 1st dose), before 3rd vaccination (8 months post 1st dose) and 2–3 months after 3rd vaccination (12 months post 1st dose). For comparison, 29 reverse transcriptase polymerase chain reaction (rt-PCR) confirmed SARS-CoV-2 infected patients (age range 24 to 84 years old, 52% female) were included comprising one outpatient and 28 hospitalized patients. Sera were collected during the early acute phase (1 day post diagnosis), convalescent phase (7 weeks post diagnosis), and 12 months after diagnosis (Fig. 1a and Table 1).

**Table 1:** The demographics of individuals enrolled in the study

	Vaccinees			Patients
	All	<80 years old	≥80 years old	All
No. of subjects	80	25	55	29^c^
Sex, M/F	32/48	9/16	23/32	14/15
Median age, years (range)	83 (27–98)	39 (27–63)	85 (80–98)	61 (24–84)
Comorbidity^a^ (%)	48 (60)	1 (4)	47 (85)	19 (66)
Immunosuppression^b^ (%)	10 (13)	0 (0)	10 (18)	13 (45)

^a^Comorbidities include chronic heart disease, chronic lung disease, chronic liver disease, chronic kidney disease, diabetes, cancer, rheumatic disease, neurological disease, and autoimmune disease.

^b^Inherent immunosuppressive disease, HIV, organ transplant, chemotherapy, Prednisone or other immunosuppressive medication.

^c^Five subjects at 12 months were excluded due to vaccination or sample missing, *n* = 24 at 12 months.

### Comparison of SARS-CoV-2 specific antibody responses across ages after COVID-19 vaccination and natural infection

Using sera from vaccinees and patients, we characterized the SARS-CoV-2 specific IgG binding to the full-length spike, S1 and S2 domains in ELISA. Neutralising antibodies in sera were measured against the infectious SARS-CoV-2 Bergen-1 strain prepared from a local clinical isolate. Due to the limited number of vaccinees and patients available at each age, mini-moving-groups consisting of individuals of the nearest 5 ages were utilized to assess antibody levels across ages in fine details with reduced influence from outliers (Fig. 1b–i, Supplementary Figures 1–4 and Supplementary Tables 1–10). After the 1st vaccination, full-length spike binding IgG was robustly elicited in all vaccinees below 90 years, with reduced potency in those above 82 years. The 2nd and 3rd vaccine doses boosted the spike binding IgG across all ages (Supplementary Tables 1 and 5). Natural infection significantly induced full-length spike binding IgG in all patients (Supplementary Tables 6 and 10). Similar patterns were observed when measuring S1 domain specific IgG (Supplementary Tables 2, 5, 7 and 10). After the 1st and 2nd vaccinations, antibodies targeting the highly conserved S2 domain were significantly boosted across all ages. Compared to the adult vaccinees, those above 90 years had significantly lower S2 domain specific IgG, which was partially alleviated after the 3rd vaccine dose. In addition, natural infection boosted S2 domain specific IgG in patents of all ages (Fig. 1b, c, Supplementary Tables 3, 5, 8 and 10).

SARS-CoV-2 neutralizing antibodies were significantly induced after vaccination or infection (Supplementary Tables 5 and 10). Titers above 40 were only observed in vaccinees below 43 years old after the 1st vaccination. The 2nd dose robustly boosted neutralizing antibody titers to 200 in vaccinees up to 80 years old. After the 3rd dose, neutralizing antibody titers above 800 were found in all vaccinees below 90 years old, whilst the oldest individuals had lower titers between 200 to 600 (Fig. 1d and Supplementary Table 4). In contrast, SARS-CoV-2 infection elicited convalescent neutralizing antibody titers to above 200 in patients of all ages (Fig. 1e and Supplementary Table 9).

We further stratified by age the magnitude of antibody responses using antibody fold-induction and the probabilities of antibody increase reaching given fold-inductions (Fig. 1f–i and Supplementary Figure 5). Overall, the probability decreased with increasing age after vaccinations, but increased with age post natural infection. Notably, vaccination elicited full-length spike binding IgG had 10 times higher fold-induction than neutralizing antibodies, while S1 domain specific IgG had 50 times higher fold-induction than S2 domain targeting IgG. Overall, antibody responses after natural infection were much more balanced (Fig. 1f–i).

**Fig. 2:**
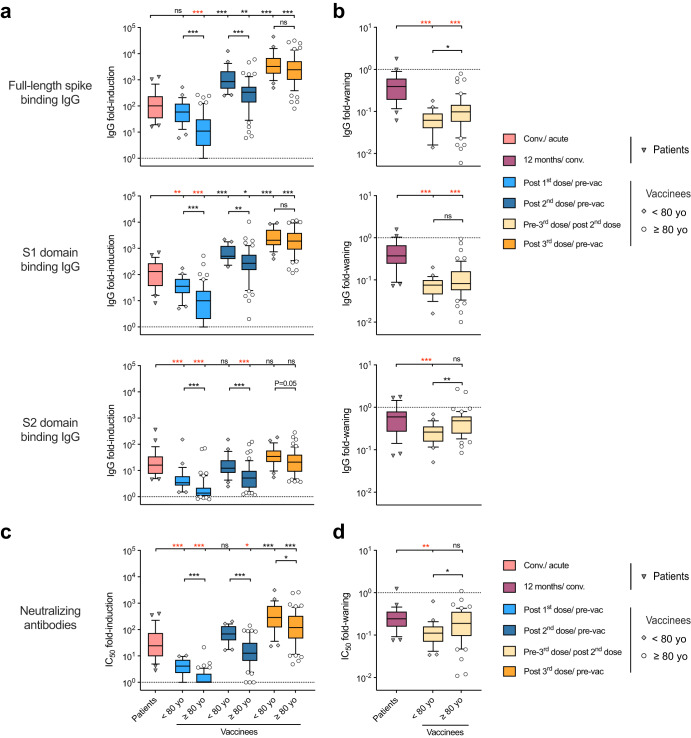
Different kinetics of homologous antibody increases in adult, elderly vaccinees and patients. **a** Fold-inductions and **b** fold-waning of SARS-CoV-2 full-length spike (top), S1 (middle) and S2 domain (bottom) binding IgG. **c** Fold-inductions and **d** fold-waning of neutralizing antibodies in the vaccinees and patients. The fold-changes are shown in boxplot, in which center line represents the median, bounds of the box represent 25–75 percentile, and whiskers represent 10–90 percentile. *P < 0.05, **P < 0.01, ***P < 0.001, ns not significant, conv. convalescent (Antibody fold-inductions and fold-wanings were Ln transformed in statistical analyses. Unpaired t test was performed between vaccinees below (*n* = 25) and above 80 years old (*n* = 55) at each time point. Ordinary one-way ANOVA and Dunnett’s multiple comparisons were performed between COVID-19 patients (*n* = 29) and the vaccinees at different time points, where * is marked in red when fold-inductions and fold-wanings were higher in the patients than vaccinees, and in black otherwise). The subjects with no detectable neutralizing antibody titer after 2nd dose were excluded in fold-waning analyses. Duplicates were performed in all experiments.

To compare the kinetics of antibody responses, vaccinees were divided into two age categories below and above 80 years old, termed adults and elderly, respectively. The 1st and 2nd vaccinations elicited significantly higher fold-induction of full-length spike, S1 and S2 domain binding IgG, as well as neutralizing antibodies, in adults than elderly. By 8 months, full-length spike and S2 domain binding IgG, as well as neutralizing antibodies waned significantly faster in adults than elderly vaccinees. Moreover, the antibody waning was overall faster in vaccinees compared to patients. The 3rd dose potently boosted antibodies in all vaccinees and largely alleviated the low antibody levels in the elderly (Fig. 2).

### COVID-19 vaccination boosted antibodies cross-reactive to endemic HCoVs

**Fig. 3:**
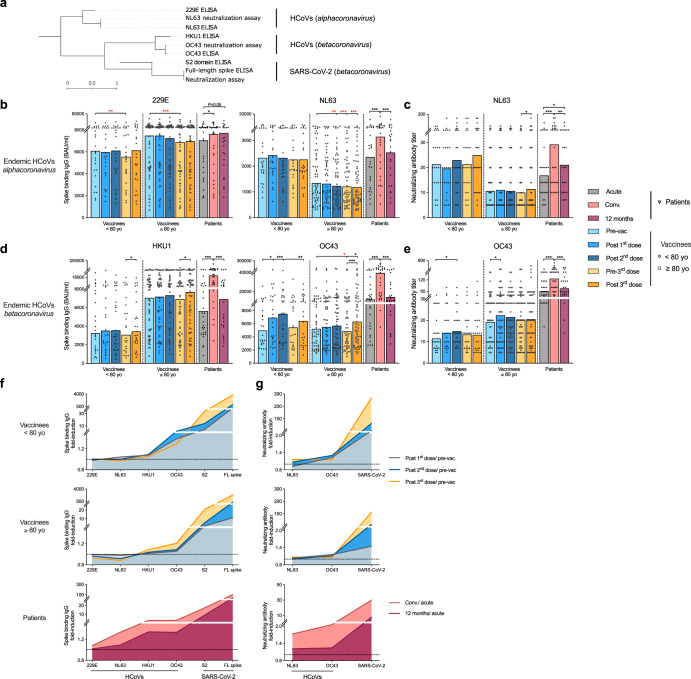
Antibody responses cross-reactive to endemic HCoV. **a** Phylogenetic tree shows the genetic divergence among spike proteins from the SARS-CoV-2 and four endemic human coronaviruses (HCoV) tested in enzyme-linked immunosorbent assay (ELISA) and neutralization assays. Phylogenetic analyses were performed at ngPhylogeny.fr. **b**, **d** Endemic HCoVs spike binding IgG concentrations in the vaccinees and patients were measured against the full-length spike proteins from *alphacoronavirus* (**b**, 229E and NL63) and *betacoronavirus* (**d**, HKU1 and OC43). **c**, **e** HCoV specific neutralizing antibody titer in the vaccinees and patients were measured against the genuine NL63 and OC43 viruses. **f**, **g** Landscape plotting of spike binding IgG (**f**) and neutralizing antibody (**g**) fold-inductions in the vaccinees below (top, *n* = 25), above 80 years old (middle, *n* = 55) and patients (bottom, *n* = 29). The geometric mean titers are shown as bars, and each symbol represents one subject in (**b**–**e**). The geometric means of fold-induction are connected as curves in (**f**, **g**). *P < 0.05, **P < 0.01, ***P < 0.001, conv. convalescent (Antibody concentrations (binding antibody unit (BAU)/ml) and titers and fold-inductions were Ln transformed in statistical analyses. RM two-way ANOVA with the Geisser-Greenhouse correction and Turkey’s multiple comparisons were performed among time points in (**b**–**e**). * is marked in red when the antibody level was lower after vaccination, and in black otherwise). The horizontal dotted lines indicate fold-induction of 1 in (**f**, **g**). Duplicates were performed in all experiments.

We measured cross-reactive IgG against the recombinant spike proteins from four endemic HCoVs in ELISA, including *alphacoronavirus* (229E and NL63) and *betacoronavirus* (HKU1 and OC43). Furthermore, we developed virus neutralization (VN) assays using infectious live NL63 and OC43 viruses (Fig. 3a). Prior to vaccination, 229E spike binding IgG was detected at similar levels between adult and elderly vaccinees, whilst NL63 binding and neutralizing antibodies were higher in adults. Significant increases of both binding and neutralizing antibodies against the *alphacoronavirus* were observed after natural infection, but not after vaccination (Fig. 3b, c).

Higher titres of pre-existing HKU-1 binding IgG were found in the elderly than adult vaccinees. Interestingly, the elderly had similar binding IgG titers but higher levels of neutralizing antibody against OC43 before vaccination compared to adults. Binding and neutralizing antibodies against *betacoronavirus* were significantly boosted in all vaccinees, although to a lesser extent in the elderly. Nonetheless, the most potent *betacoronavirus* specific antibody increases were observed in infected patients (Fig. 3d, e).

The cross-reactive antibody responses were also visualized in landscape plots. The overall magnitude of cross-reactive antibody increased across HCoV followed their genetic distances to SARS-CoV-2. Natural infection elicited the broadest increases in binding and neutralizing antibodies, which recognized all four endemic HCoVs. Vaccine-induced antibodies were only cross-reactive to the closely related OC43, and to a lesser degree HKU1 strain. Intriguingly, the HCoV cross-reactive antibodies were mainly elicited by the first two vaccinations in adults, but by the 3rd immunisation in elderly vaccinees (Fig. 3f, g).

### Association between homologous and cross-reactive antibody responses in COVID-19 patients

**Fig. 4:**
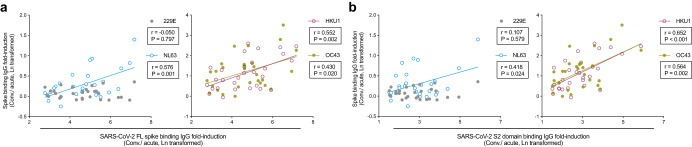
Associations between SARS-CoV-2 homologous and endemic HCoV cross-reactive antibody increases in patients. **a**, **b** Correlation between the four endemic HCoV spike binding IgG fold-inductions and the SARS-CoV-2 full-length (FL, **a**) spike and S2 domain (**b**) binding IgG increase in the patients. Each symbol represents one subject. (Spike binding IgG and neutralizing antibody fold-inductions were Ln transformed before performing Pearson correlation analyses. Linear fitting curves are plotted when Pearson *P* < 0.05. Pearson r and *P* values are noted in each correlation.).

In the patients, we analysed the relationship between homologous and cross-reactive antibody responses together with the clinical outcomes using a COVID-19 disease severity scoring system^17^ (Supplementary Table 11). No significant differences in either SARS-CoV-2 or HCoV specific antibody were found among different disease severities, albeit low number of patients (Supplementary Figure 6). Furthermore, fold inductions of the NL63, HKU1 and OC43 spike binding IgG significantly correlated with the SARS-CoV-2 full-length spike binding IgG (Fig. 4a). Notably, the HKU1 and OC43 spike binding IgG responses were more closely associated with SARS-CoV-2 S2 domain, than the full-length spike specific IgG (Fig. 4b).

### HCoV reactive antibody increases after COVID-19 vaccination correlate to S2 domain specific antibody response

**Fig. 5:**
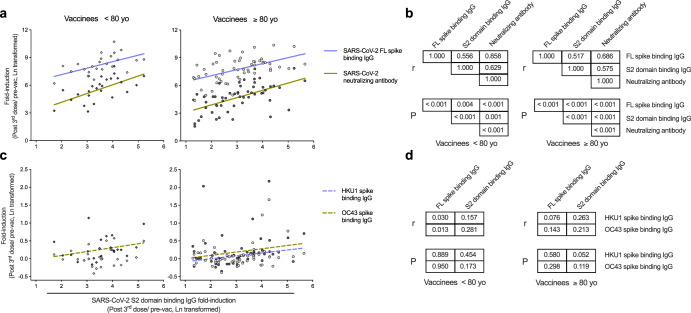
Correlations between vaccine specific and cross-reactive antibody responses in vaccinees. **a** Correlations between SARS-CoV-2 S2 domain binding IgG fold-induction and full-length (FL) spike binding IgG or neutralizing antibody fold-inductions in vaccinees below (left) and above 80 years old (right). **b** Matrix of Pearson correlation coefficients r (top) and *P* values (bottom) among SARS-CoV-2 full-length (FL) spike, S2 domain binding IgG and neutralizing antibody fold-inductions in vaccinees below (left) and above 80 years old (right). **c** Correlations between SARS-CoV-2 S2 domain binding IgG and endemic HCoV HKU1 and OC43 spike binding IgG fold-inductions in vaccinees below (left) and above 80 years old (right). **d** Matrix of Pearson correlation coefficients r (top) and *P* values (bottom) among SARS-CoV-2 full-length (FL) spike, S2 domain binding IgG and endemic HCoV HKU1 and OC43 spike binding IgG fold-inductions in vaccinees below (left) and above 80 years old (right). (IgG and neutralizing antibody fold-inductions were Ln transformed in statistical analyses. Pearson correlation analyses were performed, and linear fitting curves are plotted as solid lines when Pearson *P* < 0.05, and as dashed lines when Pearson 0.05 ≤ *P* < 0.2.

We then investigated the association between SARS-CoV-2 specific and HCoV cross-reactive antibody increases after three vaccinations. Despite the differing magnitude and kinetics, the increase in SARS-CoV-2 S2 domain targeting IgG significantly correlated with both full-length spike binding IgG and neutralizing antibody responses in both adult and elderly vaccinees (Fig. 5a, b). In contrast, no association between SARS-CoV-2 full-length spike and HCoV spike binding IgG were found in either group. The boosting in fold-inductions of *betacoronavirus* HKU1 and OC43 antibodies were in loose association, although not significant, with SARS-CoV-2 S2 domain responses in the elderly vaccinees (Fig. 5c, d).

## Discussion

Since early 2020, it has been well documented that elevated levels of cross-reactive HCoV antibodies are found after SARS-CoV-2 infection, especially in critically ill patients^7,18^. Given the sequence homology between SARS-CoV-2 and endemic HCoVs, it is not surprising that pre-existing cross-reactive antibodies are boosted by SARS-CoV-2 exposure. However, there is an open debate on whether these pre-existing pan-*(beta)coronavirus* antibodies are related to increased infection susceptibility and more severe disease^4,5^ or partial protection and favourable outcomes^6,7^. These discrepancies highlighted the challenges of investigating causal relationship between HCoV reactive antibody and disease outcomes, given the varying age, comorbidities, and disease severity of COVID-19 patients included in different retrospective clinical studies^7^. Here, we found no evidence of cross-reactive antibodies hampering SARS-CoV-2 specific responses or being associated with more severe disease in COVID-19 survivors, in agreement with studies on asymptomatic, mild and moderate SARS-CoV-2 infection^7^. Moreover, natural infection elicited the broadest increases in binding and neutralizing antibodies to SARS-CoV-2 and all four HCoVs. Vaccine-induced antibodies were less durable with a narrower repertoire, especially in the elderly, cross-reacting only with the closely related OC43, and less so with HKU1 virus.

Lower vaccine efficacy and effectiveness have been reported in elderly individuals, often attributed to immunoscenesence^19,20^. After two doses of COVID-19 vaccine, heterogeneity in immune response has been observed in both humoral and cellular compartments in different age groups^21–23^. In a large population-based cohort study on effectiveness of CoronaVac and BNT162b2 COVID-19 mass vaccination, two doses of CoronaVac reduced hospitalization, critical care admission and death in vaccinees 40–79 years of age but was less effective in older vaccinees above 80 years old. Although BNT162b2 vaccine had higher effectiveness across all ages compared to CoronaVac, a significant decline in vaccine effectiveness was observed in participants older than 80 years^24^. Hence, older individuals often have been prioritized to receive further booster doses. Several studies have found that the third immunization boosted antibodies effectively in vaccinees aged 65–90 years, which alleviated the lower antibody levels observed after the two-dose regime^25–27^. Our age stratified analysis indeed demonstrated an overwhelming hampering effect of increasing age on the SARS-CoV-2 spike S2 domain targeting IgG and neutralizing antibody responses after the first two vaccine doses, whilst the 3rd dose significantly boosted antibodies in vaccinees up to 90 years old. However, in those over 90 years old, both binding and neutralizing antibodies remained relatively low (Fig. 1). Given the significant waning of antibodies over time, it is clear that older individuals need regular boosters for protection from hospitalisation and severe disease, which highlights the urgent necessity of next-generation vaccine development.

The S2 domain of spike contains epitopes, such as the stem helix and the fusion peptide, which are highly conserved among different SARS-CoV-2 variants, and even across (sub)genera of *coronaviruses*^10,11^. The S2 domain-targeting monoclonal antibodies, S2P6 and 76E1, discovered in COVID-19 patients are broadly neutralizing^28^. New vaccine designs targeting these S2 epitopes have shown potent and broadly cross-reactive antibody responses^29–32^. Of note, the S2-HR1 domain targeting vaccine developed by Pang et al. provided in vivo protection in rhesus macaques^30^. The multivalent S2-based vaccines by Halfmann et al. offered broad protection against SARS-CoV-2 variants of concern and pangolin coronaviruses in mice and hamsters^31^. Here, we found the S2-specific IgG increase was delayed and of a lower magnitude in the elderly vaccinees (Figs. 1–2). Interestingly, elderly vaccinees had higher or similar pre-existing antibody titres to younger vaccinees against endemic *betacoronavirus*, which were further boosted after vaccination. Of note, the fold-induction of these cross-reactive antibodies correlated with the S2 domain specific IgG response (Figs. 3 and 5). In addition, natural infection elicited potent S2-specific IgG increases across all ages, which were closely associated with endemic HCoV antibody responses (Figs. 1 and 4). Our results indicate the S2 domain is a promising candidate for future vaccine designs to provide broad protection, particularly for the oldest populations.

Compared to population-based cohort studies^24,33,34^, we investigated in-depth the COVID-19 mRNA vaccine immunogenicity in SARS-CoV-2 naive individuals and compared with a group of unvaccinated COVID-19 patients (Fig. 1). Strengths of our study are the unique access to consecutive samples from cohorts with homogeneous vaccination/infection history with a wide age span which adds to the literature on distinct features of de novo and boosting immune responses in adults of all ages. Limtations of the study include that all of our vaccinees received the monovalent COVID-19 mRNA vaccine (BNT162b2), and a relatively low number of younger vaccinees and infected patients. The impact of ageing on immune responses elicited by vaccines of other platforms, such as inactivated virus or viral vector-based, is also important to investigate, but beyond the scope of current study. Furthermore, our elderly vaccinees were home-dwelling, hence in relatively good health avoiding the complications of frailties. Future studies are warranted to advance our understanding of the heterogeneity of vaccine responses in both humoral and cellular compartments, especially in high-risk groups.

Overall, we demonstrated the impact of age in the context of vaccination and natural infection on the de novo antibody responses against SARS-CoV-2 and the boosting of pre-existing endemic HCoV specific humoral immunity. Our findings emphasize the urgent need of tailored vaccine design for the elderly and suggest the S2 domain as a candidate for future universal coronavirus vaccine design.

## Methods

### Study design and participants

We conducted a prospective cohort study of adults receiving pandemic COVID-19 vaccine (BNT162b2, Pfizer BioNTech) at Eidsvåg general practice and Haukeland University Hospital in Bergen, Norway. All participants received the first two doses of BNT162b2 vaccine at a 3-week interval in January–February 2021, and the third dose of BNT162b2 vaccine 10–11 months later in November–December 2021. No vaccinee had tested rt-PCR positive for SARS-CoV-2 or had any COVID-19 symptom before receiving the 1st dose mRNA vaccine.

The COVID-19 patients were participants of a larger case-ascertained study conducted in Bergen, Norway. All patients tested rt-PCR positive for SARS-CoV-2 from nasopharyngeal swabs during March and April 2020. None of the infected patients received any COVID-19 vaccine within 12 months post diagnosis.

The study was approved by the regional ethics committee (Regional Committee for Medical Research Ethics, Western Norway (REK Vest number 118664) and Northern Norway (REK Nord number 218629)) and is registered in the National Institute for Health database Clinical trials.gov (NCT04706390). All participants provided written informed consent before inclusion in the study.

Electronic case report forms (eCRF) were used to collect demographics, comorbidities, infection history (rt-PCR test results and presence of COVID-19 symptoms), vaccination data and side reactions.

### Vaccine and sampling

The vaccine used in the study was a monovalent COVID-19 mRNA vaccine BNT162b2 embedded in lipid nanoparticles contained 30 µg of a purified single-stranded, 5’-capped messenger RNA (mRNA), encoding the viral spike protein of SARS-CoV-2 from the founder Wuhan-Hu1 strain (pre-alpha). The vaccine was supplied as a multidose vial reconstituted in sodium chloride 9 mg/mL (0.9%) containing 0.45 ml per dose, 5 doses per vial, and administered by intramuscular injection.

Serum samples were collected pre-, post 1st (day 21) and 2nd doses (2 months), and pre- (9 months) and post 3rd (12 months) vaccination from all COVID-19 vaccinees, and during the acute (0–8 days post diagnosis), convalescent phase (16–76 days post diagnosis) and 12 months (334–387 days post diagnosis) after infection from all COVID-19 patients. Sera were separated, aliquoted and stored at −80 °C until use.

### Viruses and antigens

The hCoV-19/Norway/Bergen-01/2020 (GISAID accession ID EPI_ISL_541970, termed as Bergen-1 hereafter) virus was isolated in-house from an rt-PCR-confirmed patient in March 2020 and propagated in Vero cells in a certified Biosafety Level-3 Laboratory.

The human coronavirus (HCoV) strain NL63 (GenBank: AY567487) was obtained from BEI Resources (Cat. NR-470) and propagated in LLC-MK2 cells (ATCC CCL-7) in biosafety level-2 laboratory. The HCoV strain OC43 (GenBank: AY585228) was obtained from BEI Resources (Cat. NR-52725) and propagated in HCT-8 cells (ATCC CCL-244) in biosafety level-2 laboratory.

The full-length spike proteins from SARS-CoV-2 Wuhan-1 isolate (GenBank: QHD43416), HCoV 229E strain (GenBank: A0G74783), NL63 strain (GenBank: AFV53148), HKU1 strain (UniProtKB/Swiss-Prot: Q0ZME7), and OC43 strain (GenBank: AIL49484) were produced in-house in Expi293F cells (Thermo Fisher Scientific) using the constructs provided by Prof. Barney Graham. The S1 and S2 domains of spike protein from SARS-CoV-2 Wuhan-Hu-1 isolate were obtained commercially (Sino Biological Cat. 40591-V08H and 40590-V08B, respectively).

### Enzyme-linked immunosorbent assay

To quantify the SARS-CoV-2 and HCoV spike specific binding IgG, Maxi Sorp 96-well plates (Thermo Fisher) were coated with in-house prepared full-length spike proteins (Wuhan-Hu-1 spike 0.05 μg/well; 229E, HKU1 and OC43 spikes 0.1 μg/well; NL63 spike 0.3 μg/well) or commercial spike proteins (Wuhan-Hu-1 S1 and S2 domains, 0.05 μg/well) at 4 °C overnight. Sera were 5-fold serially diluted from 1:100 and tested in duplicates. Biotin labelled anti-human IgG (1:1000, Sigma-Aldrich Cat. B-1140); horseradish peroxidase (HRP) labelled streptavidin (1:1400, Southern Biotech Cat. 7105-05) were added followed by o-Phenylenediamine dihydrochloride (OPD, 0.05 mg/well, Sigma-Aldrich Cat. P-8287). The chromogenic reaction was stopped by sulfuric acid. Optical density (OP) values were read at 490 nm using a synergy H1 plate reader (BioTek). Immunoglobulin concentrations were interpolated as binding antibody unit (BAU)/ml from the standard curve with purified human IgG (Sigma-Aldrich Cat. I-4506).

### SARS-CoV-2 microneutralization assay

To measure serum neutralizing antibody titres, the microneutralization assay was performed in a certified Biosafety Level 3 Laboratory against the infectious hCoV-19/Norway/Bergen-01/2020 (GISAID accession ID. EPI_ISL_541970). Briefly, sera were heat inactivated at 56 °C for 60 min, analysed in serial dilutions (duplicated, starting from 1:20), and mixed with 100 50% tissue culture infectious doses (TCID_50_) viruses in 96-well plates (ThermoFisher). After one hour incubation, the sera-virus mixtures were added to Vero cells and further incubated at 37 °C for 24 h. Cells were fixed and permeabilized with methanol (Sigma-Aldrich) and 0.6% H_2_O_2_ (Sigma-Aldrich) and incubated with rabbit monoclonal IgG against SARS-CoV-2 NP (1:2000, Sino Biological Cat. 40143-R019). Cells were further incubated with biotinylated goat anti-rabbit IgG (H + L) (1:2500, Southern Biotech Cat. 4050-08), and Streptavidin-HRP (1:1400, Southern Biotech Cat. 7105-05). The reactions were developed with OPD (0.05 mg/well, Sigma-Aldrich Cat. P-8287). The neutralizing (IC_50_) titer was determined as the reciprocal of the serum dilution giving 50% inhibition of virus infectivity. Negative samples were assigned a value of 10 for calculation purpose.

### HCoV virus neutralization assay

A virus neutralizing assay against the HCoV NL63 strain was developed. Serum samples were heat inactivated and 2-fold serially diluted (starting from 1:10) in DMEM supplemented with 2% heat-inactivated fetal bovine serum, 1% non-essential amino acid (Sigma-Aldrich) and 1.5 g/L sodium bicarbonate (NaHCO_3_), then incubated with 100 TCID_50_ NL63 virus at 33–34 °C for 60 min. The mixture was then added into 96-well plates (ThermoFisher) pre-seeded with LLC-MK2 cells (7000 cells/well). The virus neutralization (VN) endpoint titer against NL63 virus was determined as the highest sera dilution giving 100% inhibition of cytopathic effect on LLC-MK2 cells 7 days after infection. Negative samples were assigned a value of 5 for calculation purpose.

When testing for virus neutralizing antibodies against the HCoV OC43 strain, serum samples were heat inactivated and 2-fold serially diluted (starting from 1:10) in RPMI-1640 supplemented with 2% heat-inactivated horse serum, then incubated with 100 TCID_50_ OC43 virus at 33–34 °C for 60 min. The mixture was then added into 96-well plates (ThermoFisher) pre-seeded with HCT-8 cells (15,000 cells/well). After 13 days incubation, 100 μl/well supernatant were mixed with 50 μl human O erythrocytes (0.7% v/v). The VN endpoint titer against OC43 virus was determined as the highest sera dilution giving 100% inhibition of hemagglutination. Negative samples were assigned a value of 5 for calculation purpose.

### Phylogenetic tree

The spike protein amino acid sequences from HCoVs and SARS-CoV-2 used in ELISA and micro-/virus neutralization assays were obtained from NCBI database. Phylogenetic analyses were performed at ngPhylogeny.fr^35^ using MAFFT (Multiple Alignment using Fast Fourier Transform, default settings), BMGE (Block Mapping and Gathering with Entropy, default settings), and PhyML (Phylogeny software based on the Maximum-likelihood, default settings).

### Statistical analyses

Biological replicates were used in all experiments. Antibody titers and fold-inductions were Ln transformed prior to all statistical analyses. RM one-way or two-way ANOVA with the Geisser-Greenhouse correction and Turkey’s multiple comparisons were performed among time points within the same vaccinee or patient group. To compare adult and elderly vaccinees the unpaired t test was performed at each time point. The one-way ANOVA and Bunnett’s multiple comparisons were used to compare between COVID-19 patients and vaccinees at different time points. All statistical analyses were performed with GraphPad Prism 9.

### Reporting summary

Further information on research design is available in the Nature Research Reporting Summary linked to this article.

## Supplementary information

Supplementary material

Reporting Summary

## Data Availability

The data that support the finding of this study are available from the corresponding author upon reasonable request.

## Code availability

No custom code or mathematical algorithm that is deemed central to the conclusions were used in this study.
