# Author Correction: Impact of ageing on homologous and human-coronavirus-reactive antibodies after SARS-CoV-2 vaccination or infection

**DOI:** 10.1038/s41541-026-01388-x

**Published:** 2026-02-04

**Authors:** Fan Zhou, Juha Vahokoski, Bergen COVID-19 Research Group, Siri Øyen, Marianne Sævik, Hanne Søyland, Helene H. Sandnes, Anders Madsen, Karl A. Brokstad, Kristin G. I. Mohn, Camilla Tøndel, Nina Langeland, Rebecca J. Cox

**Affiliations:** 1 Influenza Centre, Department of Clinical Science, University of Bergen, Bergen, Norway; 2 Department of Clinical Science, University of Bergen, Bergen, Norway; 3 Department of Medicine, Haukeland University Hospitalen, Bergen, Norway; 4 Department of Microbiology, Haukeland University Hospital, Bergen, Norway; 5 Eidsvåg Family Practice, Bergen, Norway; 6 Department of Medicine, Haukeland University Hospital, Bergen, Norway; 7 Department of Safety, Chemistry and Biomedical Laboratory Sciences, Western Norway University of Applied Sciences, Bergen, Norway; 8 Department of Paediatrics, Haukeland University Hospital, Bergen, Norway

**Keywords:** Humoral immunity, RNA vaccines, Viral infection

Correction to: *npj Vaccines* 10.1038/s41541-024-00817-z, published online 20 February 2024

In this article the author name Hanne Søyland was incorrectly written as Hanne Høyland. The original article has been corrected.

