# Supplementary material for: Impact of ageing on homologous and human-coronavirus-reactive antibodies after SARS-CoV-2 vaccination or infection
Source: NPJ Vaccines. 2024 Feb 20;9:37. doi: 10.1038/s41541-024-00817-z (PMC10879087; doi:10.1038/s41541-024-00817-z)
Supplement: Supplementary file 1 — Supplementary material [file 41541_2024_817_MOESM1_ESM.pdf]

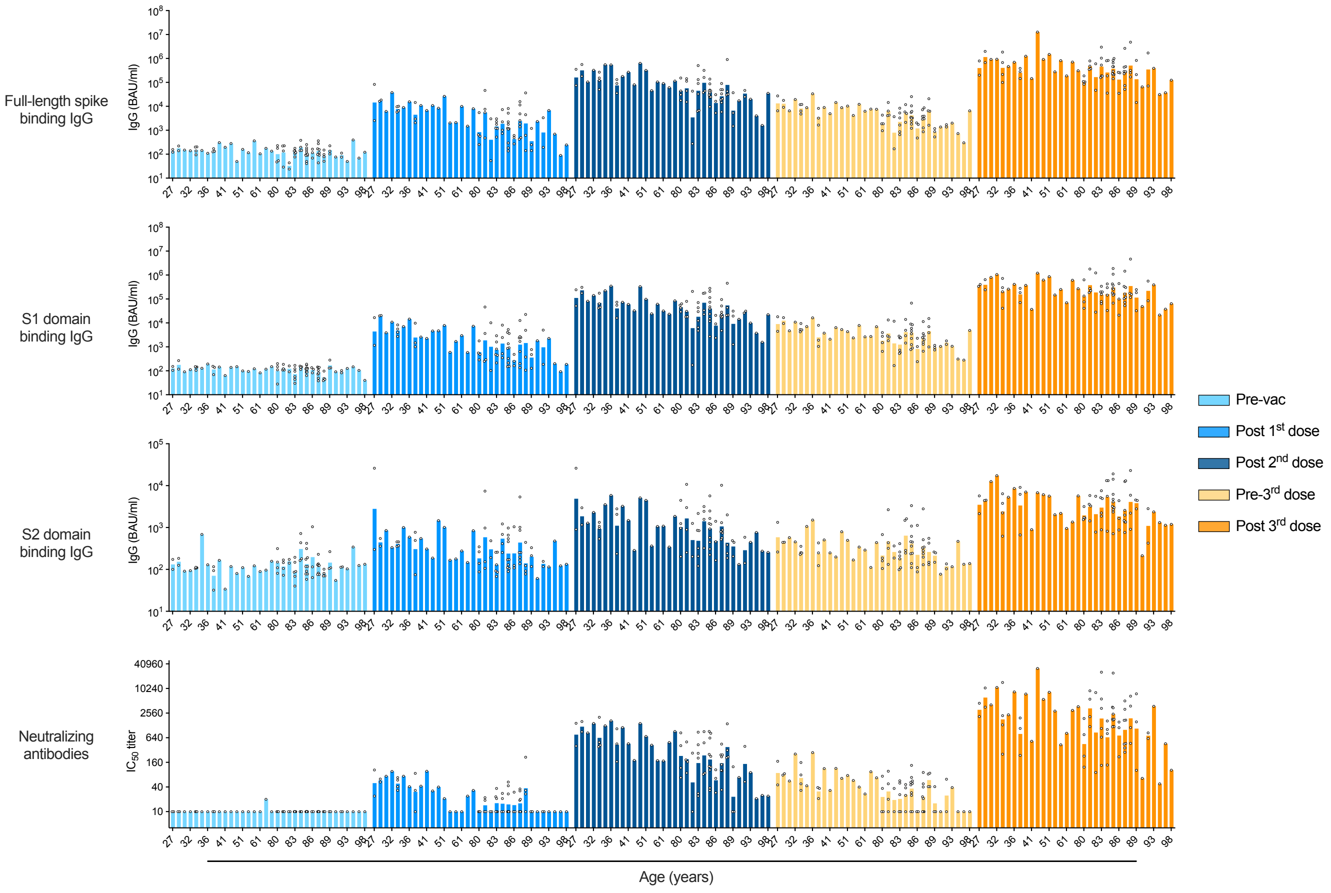

Supplementary figure 1. SARS-CoV-2 specific antibody increases after vaccination

Full-length spike, S1 and S2 domain binding IgG, and neutralizing antibody before and after vaccination were quantified. The geometric means of IgG and neutralizing antibody titers from the subjects of same age are shown as bars, and each symbol represents one subject.

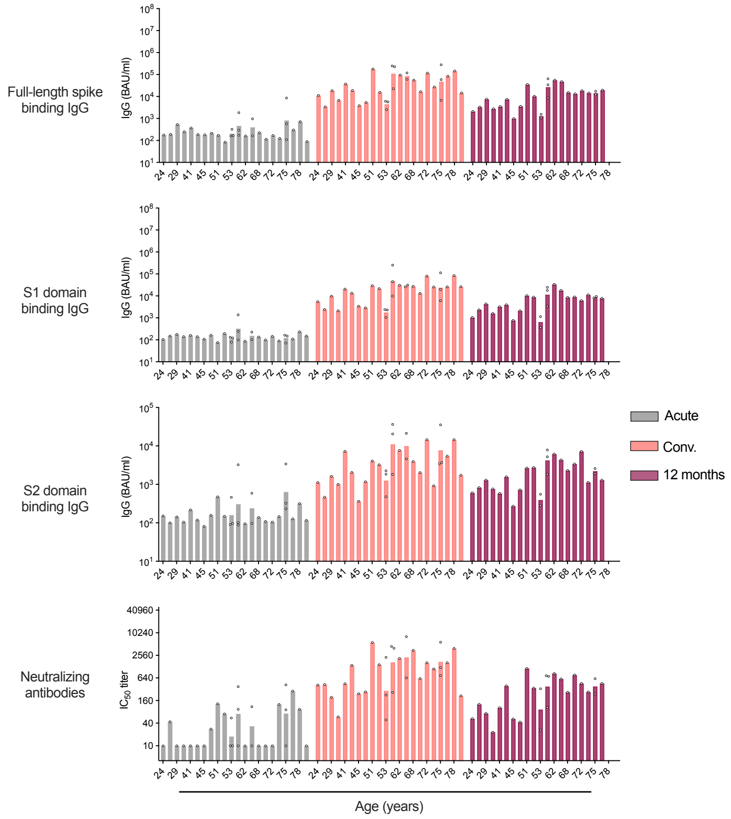

Supplementary figure 2. SARS-CoV-2 specific antibody increases after infection

Full-length spike, S1 and S2 domain binding IgG, and neutralizing antibody at acute, convalescent phase and 12 months after infection were measured. The geometric means of IgG and neutralizing antibody titers from the subjects of same age are shown as bars, and each symbol represents one subject.

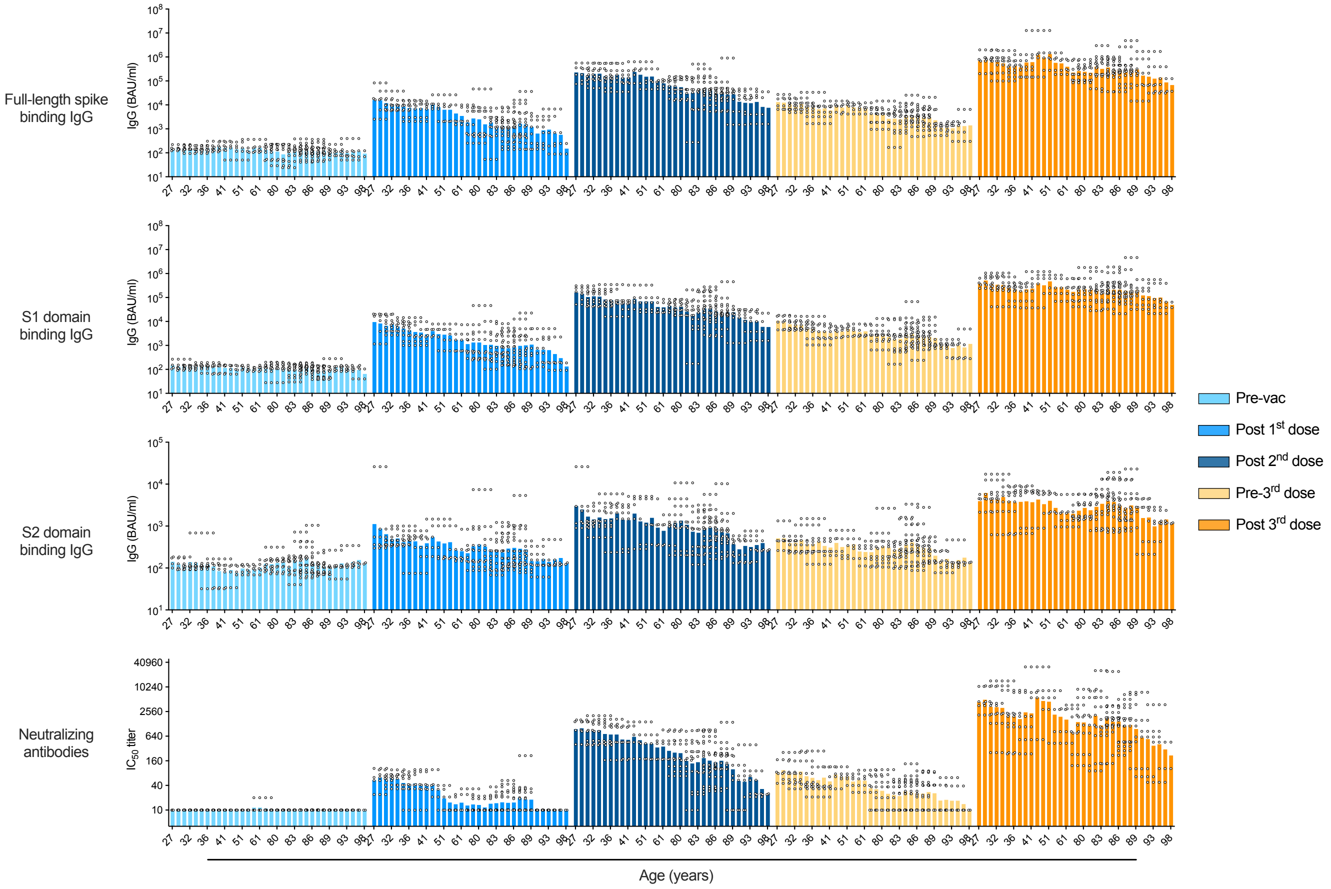

Supplementary figure 3. The impact of ageing on SARS-CoV-2 specific antibody responses after vaccination

Full-length spike, S1 and S2 domain binding IgG, and neutralizing antibody before and after vaccination were quantified. Vaccinees of the nearest 5 ages were grouped together as mini-moving-groups. Exceptions are age group 27 contains individuals aged 27 and 28; age group 28 contains individuals aged 27, 28, 29 and 32; age groups 80-90 contain individuals of the nearest 3 ages; age group 95 contains individuals aged 93, 94, 95 and 98; age group 98 contains individuals aged 95 and 98. The geometric means of IgG and neutralizing antibody titers from the subjects in the mini-moving-groups are shown as bars, and each symbol represents one subject.

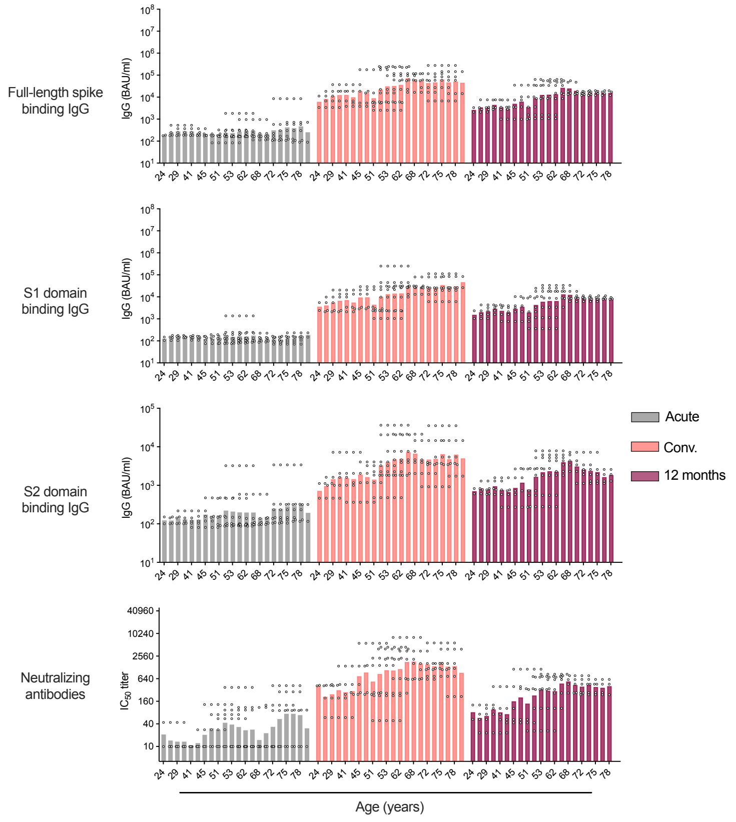

Supplementary figure 4. SARS-CoV-2 specific antibody responses after infection in patients of different ages

Full-length spike, S1 and S2 domain binding IgG, and neutralizing antibody at acute, convalescent phase and 12 months after infection were measured. Patients of the nearest 5 ages were grouped together as mini-moving-groups. Exceptions are age group 24 contains individuals aged 24 and 27; age group 27 contains individuals aged 24, 27, 29 and 35; age group 78 contains individuals aged 75, 76, 78 and 84; age group 84 contains individuals aged 78 and 84. The geometric means of IgG and neutralizing antibody titers from the subjects in the mini-moving-groups are shown as bars, and each symbol represents one subject.

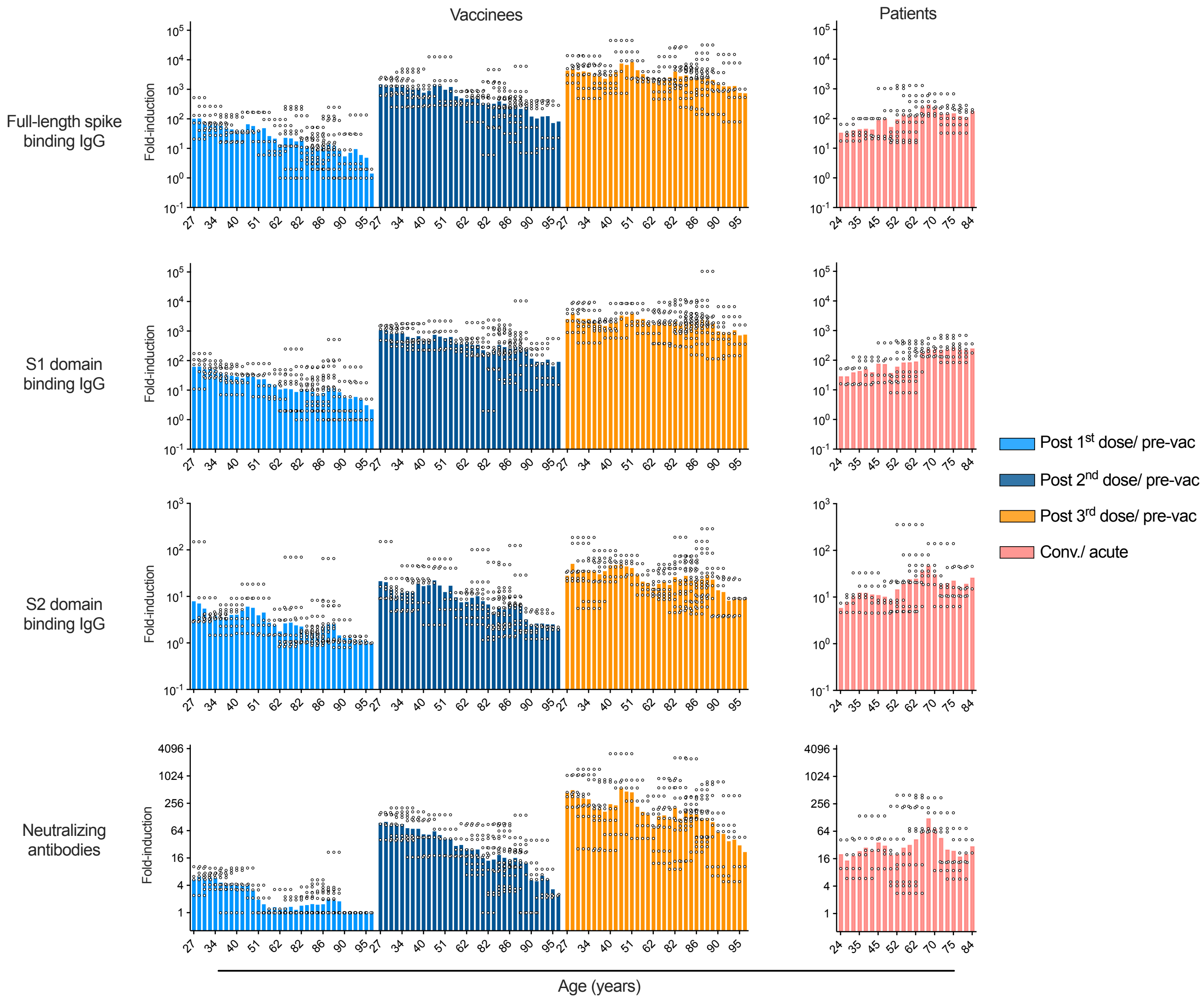

Supplementary figure 5. SARS-CoV-2 specific antibody fold-increase after vaccination or infection

The fold-increase of full-length spike, S1 and S2 domain binding IgG, and neutralizing antibody after vaccination or infection were calculated and plotted against age. Vaccinees or patients of the nearest 5 ages were grouped together as mini-moving-groups. Exceptions in vaccinees groups are age group 27 contains individuals aged 27 and 28; age group 28 contains individuals aged 27, 28, 29 and 32; age groups 80-90 contain individuals of the nearest 3 ages; age group 95 contains individuals aged 93, 94, 95 and 98; age group 98 contains individuals aged 95 and 98. Exceptions in patients groups are age group 24 contains individuals aged 24 and 27; age group 27 contains individuals aged 24, 27, 29 and 35; age group 78 contains individuals aged 75, 76, 78 and 84; age group 84 contains individuals aged 78 and 84. The geometric means of antibody fold-induction from the subjects in the mini-moving-groups are shown as bars, and each symbol represents one subject.

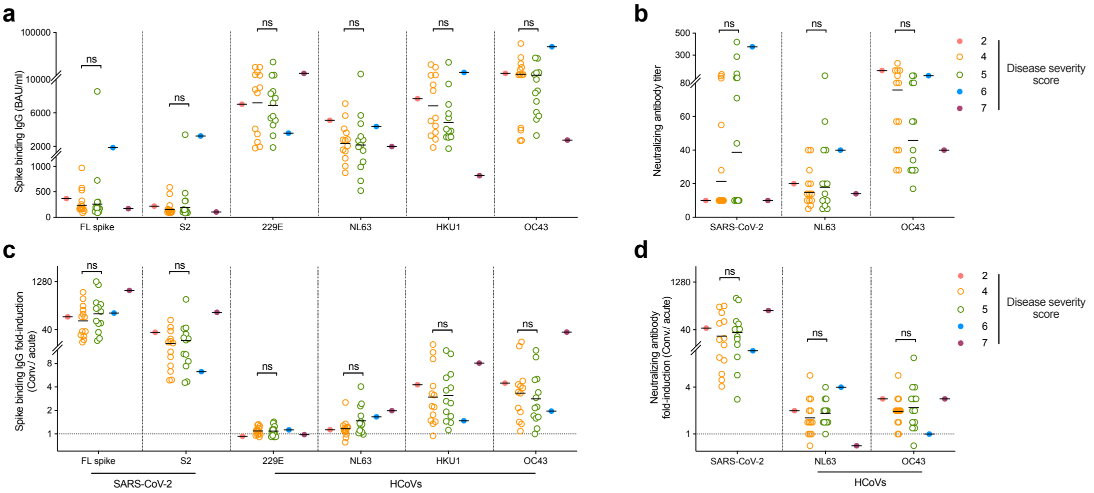

Supplementary figure 6. SARS-CoV-2 homologous and endemic HCoV cross-reactive antibody increases in patients of different severities.

(**a**-**b**) Spike binding IgG (**a**) and neutralizing antibody (**b**) at the acute phases in the patients. (**c**-**d**) Spike binding IgG (**c**) and neutralizing antibody (**d**) fold-induction between convalescent and acute phases (Conv./ acute) in the patients. COVID-19 disease severity score was modified as follows, severity 2: home-isolated with symptoms; severity 4: hospitalised with medical needs; severity 5: hospitalised needing oxygen; severity 6: hospitalised needing ventilation; severity 7: hospitalised needing respirator. The geometric means of fold-induction in each disease severity group are shown as bars, and each symbol represents one subject. ns: P>=0.05 (Antibody fold-inductions were Ln transformed, Mann-Whitney test were performed between patients of severity scores 4 and 5.) The horizontal dotted lines indicate fold-induction of 1 in **c**-**d**.

Supplementary table 1. Summary of full-length spike binding IgG titer in individuals receiving SARS-CoV-2 mRNA vaccine stratified by age

Age	N[1]	Pre-vac		Post 1st vac		Post 2nd vac		Pre-3rd vac		Post 3rd vac	
		Geomean (95% CI)	P value[2]	Geomean (95% CI)	P value	Geomean (95% CI)	P value	Geomean (95% CI)	P value	Geomean (95% CI)	P value
27-34[3]	10	152 (130-178)	Reference	11839 (6338-22114)	Reference	172978 (108471-275847)	Reference	10504 (7362-14987)	Reference	587426 (320249-1077502)	Reference
27	4	156 (122-200)	0.9998	15891 (3935-64168)	0.9996	228525 (96920-538832)	0.9994	13070 (6923-24675)	0.9993	677108 (270073-1697594)	0.9997
28	6	153 (130-179)	>0.9999	15683 (5804-42381)	0.9994	213233 (114757-396212)	0.9994	12443 (7614-20332)	0.9993	750822 (414008-1361649)	0.9993
29	10	152 (130-178)	>0.9999	11839 (6338-22114)	>0.9999	172978 (108471-275847)	>0.9999	10504 (7362-14987)	>0.9999	587426 (320249-1077502)	>0.9999
32	9	155 (130-183)	0.9999	10960 (7328-16392)	0.9997	199553 (118676-335547)	0.9995	9769 (7171-13308)	0.9996	624826 (333267-1171454)	0.9999
34	8	143 (120-172)	0.9994	10207 (6643-15683)	0.9996	200657 (111936-359701)	0.9996	10659 (6823-16652)	>0.9999	543391 (287082-1028532)	0.9998
35	10	143 (123-166)	0.9993	8391 (5012-14047)	0.9988	159074 (89152-283835)	0.9997	8040 (4690-13783)	0.9989	409293 (235680-710796)	0.9957
36	10	154 (125-191)	>0.9999	7410 (4934-11130)	0.9746	149843 (85698-261999)	0.9996	7450 (4494-12349)	0.9882	421485 (237590-747716)	0.9990
39	7	161 (125-208)	0.9996	7122 (4005-12666)	0.9842	179525 (86317-373383)	>0.9999	6683 (3313-13483)	0.9821	369711 (205837-664054)	0.9885
40	7	177 (132-238)	0.9954	7319 (4077-13137)	0.9878	136406 (70776-262894)	0.9993	7173 (3448-14925)	0.9952	592251 (186234-1883443)	>0.9999
41	7	158 (101-249)	0.9998	6712 (3925-11476)	0.9450	139501 (70179-277297)	0.9994	5919 (343110212)	0.7622	616376 (192614-1972441)	>0.9999
42	5	169 (89-319)	0.9997	11221 (7135-17645)	0.9999	239289 (123144-464979)	0.9990	9002 (6425-12611)	0.9992	1261142 (313021-5081064)	0.9903
43	5	139 (78-246)	0.9997	8017 (3559-18060)	0.9990	181985 (71258-464769)	>0.9999	7718 (4923-12102)	0.9888	934988 (205835-4247099)	0.9994
51	5	157 (79-311)	>0.9999	6351 (2404-16777)	0.9879	151086 (59359-384562)	0.9997	9257 (6083-14088)	0.9994	1319914 (385285-4521780)	0.9691
54	5	129 (69-241)	0.9994	6248 (2384-16371)	0.9836	154398 (61458-387889)	0.9997	7840 (5390-11402)	0.9866	569076 (265750-1218618)	>0.9999
57	5	166 (108-255)	0.9996	4426 (1491-13137)	0.8489	96584 (50391-185122)	0.9019	7643 (5278-11069)	0.9711	541360 (259083-1131184)	0.9998
61	5	161 (104-248)	0.9997	3491 (1623-7509)	0.3775	78905 (55940-111299)	0.2532	7204 (5117-10140)	0.8820	394408 (226087-688044)	0.9949
62	8	133 (82-215)	0.9994	1839 (724-4675)	0.0994	62918 (37739-104895)	0.1705	4015 (2171-7425)	0.2627	223619 (122929-406783)	0.4492
63	11	116 (77-177)	0.9809	2707 (1035-7078)	0.2874	58019 (35909-93743)	0.0830	428 4(2703-6791)	0.1189	260554 (165344-410588)	0.5205
80	9	112 (68-186)	0.9840	2499 (799-7811)	0.3928	54986 (30694-98501)	0.1312	3843 (2239-6596)	0.1303	241255 (145936-398832)	0.4512

81	10	86<br>(49-150)	0.6430	1544<br>(452-5277)	0.1718	29382<br>(9487-90998)	0.1963	2617<br>(1234-5551)	0.0948	218902<br>(121612-394023)	0.4029
82	12	97<br>(63-149)	0.6277	1828<br>(714-4680)	0.0775	31867<br>(10426-97401)	0.2129	2690<br>(1331-5437)	0.0653	390209<br>(214304-710500)	0.9949
83	12	115<br>(77-172)	0.9662	1278<br>(639-2556)	**0.0034**	37883<br>(11890-120698)	0.3650	2523<br>(1269-5016)	**0.0421**	319559<br>(153045-667242)	0.9746
84	20	124<br>(99-155)	0.9137	1314<br>(816-2116)	**0.0006**	48457<br>(29154-80541)	**0.0273**	3665<br>(2460-5461)	**0.0149**	367330<br>(239206-564081)	0.9755
85	17	122<br>(93-159)	0.9355	1045<br>(587-1861)	**0.0003**	39843<br>(24740-64165)	**0.0059**	3560<br>(2273-5575)	**0.0228**	284207<br>(177738-454451)	0.6843
86	21	116<br>(91-148)	0.7070	1288<br>(725-2289)	**0.0009**	28887<br>(20184-41343)	**0.0002**	3146<br>(2139-4628)	**0.0031**	287627<br>(189364-436880)	0.6545
87	15	116<br>(87-155)	0.8428	1625<br>(715-3692)	**0.0212**	31159<br>(16920-57379)	**0.0053**	3088<br>(1882-5064)	**0.0147**	282066<br>(147774-538398)	0.8353
88	15	123<br>(92-165)	0.9736	1556<br>(656-3689)	**0.0234**	26936<br>(13026-55700)	**0.0080**	2863<br>(1698-4828)	**0.0121**	284044<br>(137980-584728)	0.8938
89	9	105<br>(79-139)	0.4446	1193<br>(359-3956)	0.0909	27501<br>(8828-85673)	0.1812	2310<br>(1076-4959)	0.0705	298626<br>(97124-918184)	0.9894
90	6	112<br>(81-156)	0.8034	634<br>(212-1900)	**0.0258**	13485<br>(4689-38783)	**0.0446**	1107<br>(787-1555)	**<0.0001**	165722<br>(40407-679678)	0.8129
92	8	119<br>(75-188)	0.9901	863<br>(321-2322)	**0.0157**	12187<br>(5266-28206)	**0.0039**	1132<br>(828-1549)	**<0.0001**	149951<br>(47898-469437)	0.5544
93	6	98<br>(55-176)	0.8921	931<br>(239-3628)	0.1395	11756<br>(4272-32351)	**0.0274**	1066<br>(604-1880)	**0.0016**	124185<br>(35494-434487)	0.5066
94	6	106<br>(59-189)	0.9640	639<br>(163-2495)	0.0783	13265<br>(4537-38779)	**0.0467**	1382<br>(604-3162)	**0.0418**	138250<br>(40497-471965)	0.5679
95	4	114<br>(47-278)	0.9978	569<br>(93-3494)	0.2738	825<br>1(2042-33344)	0.1373	1306<br>(356-4793)	0.3006	86990<br>(27410-276073)	0.2926
98	2	93<br>(53-161)	0.7718	148<br>(56-396)	0.0890	7570<br>(368-155575)	0.6961	1405<br>(69-28807)	0.8785	68121<br>(20744-223698)	0.4110

[1] N indicates the number of individuals included in the corresponding mini-moving-group. Individuals of the nearest 5 ages were grouped together as mini-moving-groups. Exceptions are age group 27 contains individuals aged 27 and 28; age group 28 contains individuals aged 27, 28, 29 and 32; age groups 80-90 contain individuals of the nearest 3 ages; age group 95 contains individuals aged 93, 94, 95 and 98; age group 98 contains individuals aged 95 and 98.

[2] IgG titers were Ln transformed in statistical analyses. RM two-way ANOVA and Dunnett's multiple comparisons were performed between all the mini-moving-groups and reference group. Adjusted P values are reported here and those P<0.05 are highlighted in bold.

[3] The group consisting of 10 individuals aged 27 to 34 was used as reference in Dunnett's multiple comparisons tests.

Supplementary table 2. Summary of S1 domain binding IgG titer in individuals receiving SARS-CoV-2 mRNA vaccine stratified by age

Age	N[1]	Pre-vac		Post 1st vac		Post 2nd vac		Pre-3rd vac		Post 3rd vac	
		Geomean (95% CI)	P value[2]	Geomean (95% CI)	P value	Geomean (95% CI)	P value	Geomean (95% CI)	P value	Geomean (95% CI)	P value
27-34[3]	10	132 (109-160)	Reference	6666 (3726-11928)	Reference	105190 (66862-165490)	Reference	7691 (5466-10823)	Reference	356358 (199638-636108)	Reference
27	4	150 (99-226)	0.9994	9419 (2405-36890)	0.9995	160540 (73576-350294)	0.9919	10654 (5657-20064)	0.9925	382831 (255910-572699)	0.9998
28	6	132 (97-179)	>0.9999	8339 (3343-20802)	0.9995	140399 (81865-240787)	0.9989	9314 (5749-15088)	0.9992	512646 (327384-802747)	0.9946
29	10	132 (109-160)	>0.9999	6666 (3726-11928)	>0.9999	105190 (66862-165490)	>0.9999	7691 (5466-10823)	>0.9999	356358 (199638-636108)	>0.9999
32	9	133 (109-163)	>0.9999	7354 (4635-11669)	0.9997	113097 (70713-180885)	0.9997	7350 (5446-9919)	0.9998	341337 (178181-653889)	0.9999
34	8	130 (110-152)	0.9998	6227 (4219-9190)	0.9998	108663 (63608-185633)	>0.9999	7098 (5111-9858)	0.9996	337200 (165130-688572)	0.9999
35	10	129 (109-153)	0.9998	4933 (2856-8521)	0.9990	82999 (46865-146994)	0.9992	5414 (3251-9016)	0.9873	245057 (134338-447028)	0.9955
36	10	133 (112-157)	>0.9999	4273 (2516-7258)	0.9878	77675 (44383-135939)	0.9988	4872 (2983-7958)	0.8920	220608 (130908-371772)	0.9817
39	7	119 (88-162)	0.9993	3666 (1735-7745)	0.9695	80535 (38288-169401)	0.9993	3928 (1930-7995)	0.7901	177557 (90889-346868)	0.8664
40	7	121 (89-165)	0.9994	3452 (1675-7116)	0.9285	61051 (30472-122315)	0.9554	3878 (1917-7845)	0.7662	221120 (93378-523617)	0.9953
41	7	117 (88-155)	0.9991	2941 (1661-5205)	0.6115	60717 (30571-120589)	0.9493	3302 (1909-5712)	0.3045	234214 (95644-573542)	0.9990
42	5	115 (83-158)	0.9991	3993 (2562-6224)	0.9344	85693 (40300-182216)	0.9995	4113 (2842-5953)	0.3615	389427 (115564-1312292)	0.9999
43	5	105 (78-142)	0.9536	2975 (1235-7167)	0.8693	68640 (27661-170330)	0.9990	3747 (2435-5766)	0.3199	323883 (90565-1158288)	0.9999
51	5	120 (100-143)	0.9990	2811 (1139-6935)	0.8339	68938 (27798-170966)	0.9990	4867 (3249-7291)	0.7783	475504 (220331-1026203)	0.9993
54	5	108 (88-132)	0.9048	2573 (1075-6154)	0.7282	68824 (27715-170909)	0.9990	4073 (2637-6290)	0.4617	268490 (108972-661522)	0.9994
57	5	102 (89-118)	0.5051	1691 (640-4468)	0.4221	40550 (23416-70221)	0.2922	3572 (2310-5524)	0.2649	268230 (108960-660310)	0.9994
61	5	111 (91-136)	0.9688	1669 (644-4324)	0.3954	39318 (23787-64990)	0.2089	3925 (2359-6529)	0.5234	212739 (105060-430782)	0.9860
62	8	107 (70-161)	0.9950	1148 (538-2446)	**0.0494**	45062 (33193-61174)	0.1273	2838 (1623-4962)	0.1585	172403 (85216-348796)	0.8595
63	11	113 (82-154)	0.9957	1322 (499-3505)	0.1881	37768 (27196-52449)	**0.0429**	2836 (1788-4500)	0.0576	221105 (115276-424090)	0.9888
80	9	116 (79-169)	0.9993	1324 (409-4289)	0.3621	40511 (27530-59613)	0.0997	2870 (1622-5076)	0.1694	224196 (108914-461499)	0.9945

81	10	114 (81-161)	0.9990	1057 (322-3472)	0.2259	25733 (7920-83617)	0.4844	2275 (1025-5050)	0.2228	212183 (90812-495770)	0.9944
82	12	96 (70-131)	0.7711	1067 (387-2945)	0.1124	17828 (6419-49516)	0.1121	1820 (892-3713)	**0.0473**	210621 (110088-402961)	0.9849
83	12	98 (72-135)	0.8595	966 (432-2163)	**0.0240**	23687 (7708-72793)	0.3494	1899 (908-3968)	0.0692	157640 (85277-291409)	0.6584
84	20	104 (84-128)	0.7971	886 (517-1518)	**0.0012**	35230 (19954-62200)	0.1146	2831 (1668-4805)	0.0808	214844 (142184-324636)	0.9356
85	17	118 (103-136)	0.9950	763 (405-1438)	**0.0012**	34065 (17246-67285)	0.1924	3043 (1631-5680)	0.2544	212821 (124141-364851)	0.9715
86	21	102 (86-121)	0.5793	822 (455-1485)	**0.0011**	25701 (14961-44151)	**0.0125**	2581 (1538-4331)	**0.0367**	211638 (131895-339591)	0.9473
87	15	79 (62-102)	0.0754	959 (407-2258)	**0.0272**	23444 (12679-43349)	**0.0178**	2249 (1301-3889)	**0.0243**	190126 (97585-370422)	0.9402
88	15	87 (64-117)	0.3789	1016 (436-2368)	**0.0324**	24285 (12679-46517)	**0.0299**	2305 (1410-3766)	**0.0145**	196007 (104597-367304)	0.9471
89	9	87 (58-132)	0.7236	1077 (331-3498)	0.2374	24426 (9132-65336)	0.2686	2055 (1032-4091)	0.0875	20316 3(79143-521524)	0.9907
90	6	125 (88-177)	0.9997	657 (218-1984)	0.0882	14454 (5406-38648)	0.1018	1120 (884-1419)	**<0.0001**	122913 (52280-288974)	0.5881
92	8	128 (98-166)	0.9997	660 (262-1665)	**0.0234**	11674 (5295-25738)	**0.0104**	949 (664-1355)	**<0.0001**	114410 (49899-262325)	0.4705
93	6	107 (90-127)	0.8165	645 (173-2401)	0.1686	9428 (3734-23809)	**0.0300**	780 (420-1449)	**0.0035**	95318 (33283-272976)	0.5210
94	6	93 (65-135)	0.7944	441 (121-1610)	0.0858	10150 (3877-26569)	**0.0430**	1006 (423-2390)	0.0516	99749 (35475-280476)	0.5452
95	4	94 (53-167)	0.9649	298 (76-1164)	0.1149	6032 (1955-18611)	0.0832	821 (223-3018)	0.2568	67484 (19679-231414)	0.4553
98	2	64 (25-165)	0.8321	132 (66-264)	**0.0295**	5915 (442-79125)	0.6637	1169 (72-18966)	0.8736	49356 (29109-83687)	0.0619

[1] N indicates the number of individuals included in the corresponding mini-moving-group. Individuals of the nearest 5 ages were grouped together as mini-moving-groups. Exceptions are age group 27 contains individuals aged 27 and 28; age group 28 contains individuals aged 27, 28, 29 and 32; age groups 80-90 contain individuals of the nearest 3 ages; age group 95 contains individuals aged 93, 94, 95 and 98; age group 98 contains individuals aged 95 and 98.

[2] IgG titers were Ln transformed in statistical analyses. RM two-way ANOVA and Dunnett's multiple comparisons were performed between all the mini-moving-groups and reference group. Adjusted P values are reported here and those P<0.05 are highlighted in bold.

[3] The group consisting of 10 individuals aged 27 to 34 was used as reference in Dunnett's multiple comparisons tests.

Supplementary table 3. Summary of S2 domain binding IgG titer in individuals receiving SARS-CoV-2 mRNA vaccine stratified by age

Age	N[1]	Pre-vac		Post 1st vac		Post 2nd vac		Pre-3rd vac		Post 3rd vac	
		Geomean (95% CI)	P value[2]	Geomean (95% CI)	P value	Geomean (95% CI)	P value	Geomean (95% CI)	P value	Geomean (95% CI)	P value
27-34[3]	10	117 (101-136)	Reference	642 (280-1468)	Reference	1678 (861-3271)	Reference	395 (284-550)	Reference	4227 (2356-7583)	Reference
27	4	142 (107-187)	0.9608	1116 (140-8865)	0.9995	3005 (669-13497)	0.9972	512 (262-1001)	0.9972	3957 (2618-5980)	0.9998
28	6	123 (96-158)	0.9996	871 (222-3419)	0.9996	2493 (927-6701)	0.9992	513 (334-787)	0.9949	6142 (3337-11304)	0.9955
29	10	117 (101-136)	>0.9999	642 (280-1468)	>0.9999	1678 (861-3271)	>0.9999	395 (284-550)	>0.9999	4227 (2356-7583)	>0.9999
32	9	139 (92-210)	0.9990	486 (374-631)	0.9992	1440 (976-2124)	0.9996	404 (293-555)	>0.9999	4521 (2404-8500)	0.9998
34	8	135 (85-213)	0.9993	510 (383-679)	0.9994	1609 (945-2741)	0.9999	465 (285-756)	0.9993	4914 (2361-10225)	0.9996
35	10	115 (73-182)	>0.9999	415 (270-639)	0.9952	1473 (820-2645)	0.9997	371 (233-591)	0.9997	3993 (2017-7903)	0.9999
36	10	122 (77-193)	0.9998	437 (284-673)	0.9989	1524 (836-2778)	0.9997	375 (235-599)	0.9998	3651 (1964-6786)	0.9996
39	7	108 (50-233)	0.9998	434 (230-819)	0.9990	2017 (935-4351)	0.9996	429 (224-821)	0.9997	3782 (1671-8558)	0.9997
40	7	84 (52-137)	0.9468	342 (187-625)	0.9781	1409 (567-3500)	0.9996	337 (185-617)	0.9994	3915 (1710-8966)	0.9998
41	7	78 (49-125)	0.8223	389 (189-800)	0.9953	1383 (567-3375)	0.9996	308 (193-492)	0.9957	3730 (1669-8336)	0.9997
42	5	90 (53-152)	0.9908	544 (261-1138)	0.9997	1997 (705-5659)	0.9997	398 (241-656)	>0.9999	4309 (1985-9354)	>0.9999
43	5	75 (49-117)	0.6789	429 (180-1023)	0.9991	129 2(394-4239)	0.9996	318 (179-567)	0.9993	3350 (1542-7280)	0.9994
51	5	97 (78-122)	0.9257	384 (151-978)	0.9990	1209 (369-3964)	0.9994	340 (193-597)	0.9995	4007 (2381-6743)	0.9999
54	5	92 (75-113)	0.6848	415 (171-1009)	0.9991	1578 (597-4172)	0.9999	367 (220-612)	0.9997	2710 (1372-5352)	0.9909
57	5	96 (79-116)	0.8160	262 (131-526)	0.8234	917 (366-2298)	0.9890	248 (147-417)	0.8749	2007 (1120-3595)	0.7375
61	5	103 (78-136)	0.9990	252 (135-473)	0.7441	769 (401-1478)	0.8127	241 (148-393)	0.8024	2012 (1118-3618)	0.7446
62	8	128 (94-173)	0.9994	226 (140-366)	0.5071	974 (502-1888)	0.9868	239 (151-378)	0.7543	1978 (1351-2898)	0.5061
63	11	124 (97-159)	0.9995	327 (152-705)	0.9857	1169 (569-2402)	0.9990	296 (169-519)	0.9956	2322 (1620-3328)	0.7763
80	9	133 (99-177)	0.9990	364 (144-920)	0.9954	1352 (585-3123)	0.9995	330 (172-635)	0.9994	2718 (1897-3894)	0.9676

81	10	135<br>(104-175)	0.9952	323<br>(142-733)	0.9863	1074<br>(469-2463)	0.9989	307<br>(170-554)	0.9990	2395<br>(1602-3581)	0.8570
82	12	109<br>(83-143)	0.9994	247<br>(117-520)	0.7985	732<br>(348-1542)	0.8315	270<br>(164-443)	0.9735	2868<br>(1864-4414)	0.9895
83	12	151<br>(97-234)	0.9887	241<br>(146-397)	0.5981	695<br>(371-1302)	0.6635	299<br>(182-491)	0.9951	3413<br>(1907-6110)	0.9994
84	20	142<br>(103-197)	0.9937	235<br>(159-347)	0.4983	837<br>(530-1322)	0.7939	357<br>(234-544)	0.9996	3942<br>(2604-5968)	0.9998
85	17	176<br>(117-263)	0.6967	292<br>(188-452)	0.8110	921<br>(570-1486)	0.9209	411<br>(248-682)	0.9999	3802<br>(2350-6149)	0.9997
86	21	134<br>(99-180)	0.9990	303<br>(191-478)	0.8610	897<br>(592-1360)	0.8617	359<br>(234-549)	0.9996	3061<br>(2020-4638)	0.9955
87	15	116<br>(81-165)	>0.9999	288<br>(158-523)	0.8743	714<br>(424-1203)	0.6001	299<br>(187-480)	0.9949	2707<br>(1576-4649)	0.9882
88	15	109<br>(89-134)	0.9993	277<br>(158-486)	0.8166	672<br>(405-1114)	0.4917	297<br>(200-440)	0.9884	3131<br>(1873-5236)	0.9990
89	9	97<br>(72-131)	0.9872	146<br>(95-225)	0.1217	367<br>(216-623)	0.0515	198<br>(131-298)	0.2696	2760<br>(1184-6433)	0.9989
90	6	114<br>(76-171)	0.9999	145<br>(91-230)	0.1291	280<br>(165-476)	**0.0199**	148<br>(100-217)	**0.0448**	1557<br>(545-4447)	0.8095
92	8	129<br>(86-194)	0.9994	164<br>(104-258)	0.1876	337<br>(212-536)	**0.0278**	166<br>(110-249)	0.0931	1608<br>(740-3498)	0.6250
93	6	121<br>(75-195)	0.9999	140<br>(81-241)	0.1403	318<br>(176-573)	**0.0461**	141<br>(85-232)	0.1048	984<br>(440-2199)	0.2027
94	6	141<br>(98-201)	0.9918	160<br>(103-248)	0.1718	355<br>(216-582)	**0.0446**	155<br>(100-241)	0.1048	1312<br>(768-2242)	0.1724
95	4	156<br>(92-265)	0.9777	173<br>(89-339)	0.3719	394<br>(239-648)	0.0844	178<br>(94-336)	0.5366	1439<br>(1032-2007)	0.1235
98	2	129<br>(121-139)	0.9735	128<br>(117-140)	0.0590	265<br>(248-283)	**0.0069**	135<br>(131-140)	**0.0023**	1167<br>(1109-1227)	**0.0297**

[1] N indicates the number of individuals included in the corresponding mini-moving-group. Individuals of the nearest 5 ages were grouped together as mini-moving-groups. Exceptions are age group 27 contains individuals aged 27 and 28; age group 28 contains individuals aged 27, 28, 29 and 32; age groups 80-90 contain individuals of the nearest 3 ages; age group 95 contains individuals aged 93, 94, 95 and 98; age group 98 contains individuals aged 95 and 98.

[2] IgG titers were Ln transformed in statistical analyses. RM two-way ANOVA and Dunnett's multiple comparisons were performed between all the mini-moving-groups and reference group. Adjusted P values are reported here and those P<0.05 are highlighted in bold.

[3] The group consisting of 10 individuals aged 27 to 34 was used as reference in Dunnett's multiple comparisons tests.

Supplementary table 4. Summary of neutralizing antibody titer in individuals receiving SARS-CoV-2 mRNA vaccine stratified by age

Age	N[1]	Pre-vac		Post 1st vac		Post 2nd vac		Pre-3rd vac		Post 3rd vac	
		Geomean (95% CI)	P value[2]	Geomean (95% CI)	P value	Geomean (95% CI)	P value	Geomean (95% CI)	P value	Geomean (95% CI)	P value
27-34[3]	10	10 (10-10)	Reference	56.33 (42.36-74.9)	Reference	836.01 (562.62-1242.25)	Reference	82.47 (54.84-124.02)	Reference	3337.74 (1591.54-6999.83)	Reference
27	4	10 (10-10)	n.a.	53.73 (29.59-97.55)	0.9999	953.65 (513.94-1769.56)	0.9996	84.51 (49.4-144.57)	>0.9999	4353.05 (2260.43-8382.91)	0.9994
28	6	10 (10-10)	n.a.	62.43 (40.79-95.56)	0.9996	1000.48 (658.97-1518.98)	0.9992	94.92 (55.71-161.72)	0.9995	5019.19 (3002.73-8389.78)	0.9954
29	10	10 (10-10)	n.a.	56.33 (42.36-74.9)	>0.9999	836.01 (562.62-1242.25)	>0.9999	82.47 (54.84-124.02)	>0.9999	3337.74 (1591.54-6999.83)	>0.9999
32	9	10 (10-10)	n.a.	59.63 (48.22-73.73)	0.9996	892.66 (600-1328.08)	0.9997	75.67 (49.55-115.55)	0.9997	3267.4 (1435.88-7435.07)	>0.9999
34	8	10 (10-10)	n.a.	57.35 (44.39-74.09)	>0.9999	897.36 (569.72-1413.43)	0.9997	87.38 (49.04-155.68)	0.9998	3144.3 (1259.65-7848.71)	0.9999
35	10	10 (10-10)	n.a.	46.26 (30.68-69.74)	0.9990	724.54 (439.22-1195.2)	0.9994	67.06 (38.25-117.57)	0.9993	2024.73 (837.32-4895.98)	0.9958
36	10	10 (10-10)	n.a.	42.59 (29.17-62.19)	0.9861	707.74 (434.08-1153.92)	0.9994	61.79 (37.57-101.62)	0.9955	1951.03 (829.39-4589.5)	0.9950
39	7	10 (10-10)	n.a.	44.6 (25.07-79.34)	0.9991	705.55 (387.85-1283.5)	0.9994	54.04 (28.14-103.77)	0.9883	1689.17 (637.04-4478.98)	0.9880
40	7	10 (10-10)	n.a.	39.56 (22.69-68.99)	0.9858	534.68 (273.54-1045.16)	0.9851	62.12 (31.54-122.34)	0.9991	2449.17 (681.11-8806.78)	0.9995
41	7	10 (10-10)	n.a.	39.42 (22.61-68.74)	0.9837	523.17 (273.77-999.79)	0.9711	50.5 (31.33-81.38)	0.8713	2305.77 (662.46-8025.52)	0.9994
42	5	10 (10-10)	n.a.	40.54 (24.83-66.19)	0.9754	620.7 (302.38-1274.1)	0.9991	73.38 (46.97-114.64)	0.9996	5658 (1539.13-20799.34)	0.9991
43	5	10 (10-10)	n.a.	30.43 (14.62-63.33)	0.8422	509.26 (262.7-987.26)	0.9503	64.22 (43.22-95.41)	0.9956	4670.61 (1252.55-17416.16)	0.9995
51	5	10 (10-10)	n.a.	19.31 (10.98-33.96)	0.1603	421.37 (192.48-922.45)	0.8493	66.74 (47.67-93.43)	0.9990	4488.57 (1129.06-17844.37)	0.9996
54	5	10 (10-10)	n.a.	15.31 (8.84-26.49)	0.0658	418.52 (189.82-922.79)	0.8473	50.03 (34.61-72.32)	0.7304	2164.07 (719.29-6510.85)	0.9992
57	5	11.49 (8.75-15.07)	n.a.	13.82 (9.35-20.42)	0.0063	338.19 (196.06-583.37)	0.3011	53.98 (34.67-84.04)	0.9261	1910.39 (686.96-5312.64)	0.9956
61	5	11.49 (8.75-15.07)	n.a.	15.13 (9.12-25.1)	0.0435	357.89 (192.16-666.53)	0.4736	52.5 (34.34-80.24)	0.8780	1625.86 (708.18-3732.69)	0.9582
62	8	10.91 (9.2-12.92)	n.a.	12.95 (9.25-18.14)	0.0003	280.74 (145.84-540.43)	0.2120	34.22 (18.83-62.2)	0.3723	795.67 (343.18-1844.75)	0.3018
63	11	10.65 (9.41-12.05)	n.a.	13.75 (10.48-18.04)	<0.0001	254.95 (149.87-433.72)	0.0465	32.57 (19.1-55.54)	0.2073	1419.52 (656.1-3071.21)	0.8622
80	9	10 (10-10)	n.a.	13.39 (9.91-18.08)	0.0001	247.27 (131.37-465.42)	0.1030	29.53 (16.11-54.14)	0.2093	1388.06 (547.85-3516.84)	0.9126

81	10	10 (10-10)	n.a.	11.54 (9.56-13.93)	**<0.0001**	158.89 (73.08-345.49)	**0.0417**	24.97 (14.37-43.4)	0.0612	1143.43 (403.57-3239.67)	0.8173
82	12	10 (10-10)	n.a.	14.33 (10.97-18.7)	**<0.0001**	138.84 (64.41-299.25)	**0.0174**	23.29 (14.48-37.47)	**0.0167**	2014.74 (768.94-5278.98)	0.9989
83	12	10 (10-10)	n.a.	14.74 (11.1-19.57)	**<0.0001**	148.89 (64.8-342.08)	**0.0395**	21.87 (14.48-33.04)	**0.0053**	1170.41 (437.29-3132.62)	0.8062
84	20	10 (10-10)	n.a.	15.51 (11.97-20.08)	**<0.0001**	185.83 (108.92-317.02)	**0.0033**	28.67 (20.16-40.76)	**0.0193**	1740.41 (979.97-3090.94)	0.9474
85	17	10 (10-10)	n.a.	15.09 (11.32-20.1)	**<0.0001**	161.54 (92.83-281.09)	**0.0019**	26.8 (17.95-40.02)	**0.0179**	1444.55 (851.78-2449.83)	0.7182
86	21	10 (10-10)	n.a.	15.35 (11.89-19.82)	**<0.0001**	146.76 (94.78-227.25)	**0.0001**	25.57 (17.67-37.01)	**0.0086**	1460.76 (921.06-2316.7)	0.6838
87	15	10 (10-10)	n.a.	19.65 (12.79-30.19)	**0.0129**	160.11 (97.2-263.72)	**0.0010**	25.16 (16.11-39.3)	**0.0181**	1118.37 (642.7-1946.08)	0.3870
88	15	10 (10-10)	n.a.	18.26 (11.84-28.17)	**0.0073**	133.09 (68.01-260.47)	**0.0034**	27.64 (17.97-42.51)	**0.0311**	1208.88 (628.14-2326.57)	0.5747
89	9	10 (10-10)	n.a.	17.93 (9.05-35.52)	0.1569	99.39 (34.8-283.83)	0.0579	25.75 (13.93-47.61)	0.1204	956.03 (314.02-2910.58)	0.6994
90	6	10 (10-10)	n.a.	10 (10-10)	**<0.0001**	51.38 (16.21-162.9)	**0.0464**	17.15 (8.72-33.73)	0.0557	587.55 (143.11-2412.25)	0.5317
92	8	10 (10-10)	n.a.	10 (10-10)	**<0.0001**	49.35 (20.29-120)	**0.0039**	17.76 (10.17-31.02)	**0.0138**	542.12 (145.34-2022.18)	0.3936
93	6	10 (10-10)	n.a.	10 (10-10)	**<0.0001**	64.25 (27.55-149.79)	**0.0133**	17 (8.71-33.18)	0.0510	375.46 (100.46-1403.25)	0.2364
94	6	10 (10-10)	n.a.	10 (10-10)	**<0.0001**	53.88 (21.83-132.98)	**0.0137**	17 (8.71-33.18)	0.0510	405.41 (116.55-1410.18)	0.2266
95	4	10 (10-10)	n.a.	10 (10-10)	**<0.0001**	32.72 (16.71-64.09)	**0.0043**	14.05 (7.21-27.38)	0.0605	304.29 (46.2-2004)	0.4944
98	2	10 (10-10)	n.a.	10 (10-10)	**<0.0001**	24.49 (23.53-25.49)	**<0.0001**	10 (10-10)	**<0.0001**	217.43 (50.27-940.42)	0.3970

[1] N indicates the number of individuals included in the corresponding mini-moving-group. Individuals of the nearest 5 ages were grouped together as mini-moving-groups. Exceptions are age group 27 contains individuals aged 27 and 28; age group 28 contains individuals aged 27, 28, 29 and 32; age groups 80-90 contain individuals of the nearest 3 ages; age group 95 contains individuals aged 93, 94, 95 and 98; age group 98 contains individuals aged 95 and 98.

[2] Neutralizing antibody titers were Ln transformed in statistical analyses. RM two-way ANOVA and Dunnett's multiple comparisons were performed between all the mini-moving-groups and reference group. Adjusted P values are reported here and those P<0.05 are highlighted in bold.

[3] The group consisting of 10 individuals aged 27 to 34 was used as reference in Dunnett's multiple comparisons tests.

Supplementary table 5. Summary of antibody responses in individuals receiving SARS-CoV-2 mRNA vaccine stratified by age

Age	N[1]	Full-length spike binding IgG			S1 domain binding IgG			S2 domain binding IgG			Neutralizing antibody		
		Post 1st dose[2]	Post 2nd dose	Post 3rd dose	Post 1st dose	Post 2nd dose	Post 3rd dose	Post 1st dose	Post 2nd dose	Post 3rd dose	Post 1st dose	Post 2nd dose	Post 3rd dose
27	4	0.0158	0.0005	0.0009	0.0158	0.0003	0.0007	0.2898	0.0491	0.0003	0.0294	0.0018	0.001
28	6	0.0007	<0.0001	<0.0001	0.0004	<0.0001	<0.0001	0.0767	0.0027	0.0004	0.0012	<0.0001	<0.0001
29	10	<0.0001	<0.0001	<0.0001	<0.0001	<0.0001	<0.0001	0.0054	<0.0001	<0.0001	<0.0001	<0.0001	<0.0001
32	9	<0.0001	<0.0001	<0.0001	<0.0001	<0.0001	<0.0001	0.0002	<0.0001	<0.0001	<0.0001	<0.0001	<0.0001
34	8	<0.0001	<0.0001	<0.0001	<0.0001	<0.0001	<0.0001	0.0005	0.0001	0.0003	<0.0001	<0.0001	<0.0001
35	10	<0.0001	<0.0001	<0.0001	<0.0001	<0.0001	<0.0001	<0.0001	<0.0001	<0.0001	0.0002	<0.0001	<0.0001
36	10	<0.0001	<0.0001	<0.0001	<0.0001	<0.0001	<0.0001	<0.0001	<0.0001	<0.0001	0.0001	<0.0001	<0.0001
39	7	<0.0001	<0.0001	<0.0001	0.0001	<0.0001	<0.0001	0.0033	0.0003	0.0001	0.0069	<0.0001	0.0002
40	7	<0.0001	<0.0001	<0.0001	0.0001	<0.0001	<0.0001	0.0026	0.0012	<0.0001	0.0087	<0.0001	0.0005
41	7	0.0001	<0.0001	<0.0001	<0.0001	<0.0001	<0.0001	0.0069	0.0015	<0.0001	0.0088	<0.0001	0.0005
42	5	0.001	0.001	0.0006	0.0003	0.0002	0.0002	0.0371	0.0167	<0.0001	0.0138	0.001	0.0019
43	5	0.0024	0.0013	0.0008	0.0035	0.0003	0.0003	0.0506	0.0331	0.0001	0.1069	0.0009	0.0022
51	5	0.0131	0.0021	0.0003	0.0051	0.0003	<0.0001	0.1364	0.0406	0.0004	0.2104	0.002	0.0027
54	5	0.0124	0.0013	0.0002	0.0051	0.0002	0.0001	0.0821	0.0106	0.0018	0.4575	0.0021	0.0019
57	5	0.0211	0.0002	<0.0001	0.016	<0.0001	0.0001	0.1033	0.0156	0.0011	0.5719	0.0006	0.0016
61	5	0.0131	<0.0001	<0.0001	0.0111	<0.0001	<0.0001	0.0543	0.0031	0.0005	0.6264	0.0011	0.0007
62	8	0.0037	<0.0001	<0.0001	0.0026	<0.0001	<0.0001	0.1303	0.0002	<0.0001	0.6211	<0.0001	<0.0001
63	11	0.0005	<0.0001	<0.0001	0.0016	<0.0001	<0.0001	0.0961	0.0001	<0.0001	0.2032	<0.0001	<0.0001
80	9	0.0035	<0.0001	<0.0001	0.0091	<0.0001	<0.0001	0.1844	0.0007	<0.0001	0.252	<0.0001	<0.0001
81	10	0.0052	<0.0001	<0.0001	0.0119	<0.0001	<0.0001	0.2001	0.0016	<0.0001	0.4243	0.0002	<0.0001
82	12	0.0004	<0.0001	<0.0001	0.0009	<0.0001	<0.0001	0.1143	0.0024	<0.0001	0.0702	0.0001	<0.0001
83	12	0.0002	<0.0001	<0.0001	0.0002	<0.0001	<0.0001	0.0025	0.0018	<0.0001	0.0659	0.0002	<0.0001
84	20	<0.0001	<0.0001	<0.0001	<0.0001	<0.0001	<0.0001	0.0011	<0.0001	<0.0001	0.0123	<0.0001	<0.0001
85	17	<0.0001	<0.0001	<0.0001	<0.0001	<0.0001	<0.0001	0.0054	<0.0001	<0.0001	0.0413	<0.0001	<0.0001
86	21	<0.0001	<0.0001	<0.0001	<0.0001	<0.0001	<0.0001	0.0057	<0.0001	<0.0001	0.0127	<0.0001	<0.0001
87	15	<0.0001	<0.0001	<0.0001	0.0006	<0.0001	<0.0001	0.0277	0.0001	<0.0001	0.0266	<0.0001	<0.0001
88	15	0.0003	<0.0001	<0.0001	0.0008	<0.0001	<0.0001	0.0254	<0.0001	<0.0001	0.0525	<0.0001	<0.0001
89	9	0.0161	<0.0001	<0.0001	0.027	<0.0001	<0.0001	0.2619	0.0067	0.0002	0.341	0.0085	0.0002
90	6	0.0983	0.0022	0.0003	0.1423	0.0011	0.0002	0.5957	0.0356	0.0047	n.a.	0.0882	0.0058
92	8	0.0475	0.0004	<0.0001	0.0557	<0.0001	<0.0001	0.371	0.0044	0.0007	n.a.	0.0239	0.0015
93	6	0.0946	0.0024	0.0008	0.1417	0.0011	0.0002	0.1691	0.0179	0.007	n.a.	0.0181	0.0071

94	6	0.1808	**0.0025**	**0.0008**	0.1789	**0.0017**	**0.0002**	0.2765	**0.0237**	**0.0036**	n.a.	**0.0343**	**0.0051**
95	4	0.5111	**0.0461**	**0.0152**	0.4058	**0.0342**	**0.0064**	0.5881	**0.038**	**0.0226**	n.a.	0.085	0.0796
98	2	0.4613	0.3011	0.0538	0.8177	0.4048	0.1223	0.7673	0.1045	**0.0052**	n.a.	**0.0198**	0.2062

[1] N indicates the number of individuals included in the corresponding mini-moving-group. Individuals of the nearest 5 ages were grouped together as mini-moving-groups. Exceptions are age group 27 contains individuals aged 27 and 28; age group 28 contains individuals aged 27, 28, 29 and 32; age groups 80-90 contain individuals of the nearest 3 ages; age group 95 contains individuals aged 93, 94, 95 and 98; age group 98 contains individuals aged 95 and 98.

[2] IgG titers and neutralizing antibody titers were Ln transformed in statistical analyses. RM two-way ANOVA and Dunnett's multiple comparisons were performed between pre- and post-vaccination time points for all the mini-moving-groups. Adjusted P values are reported here and those $P<0.05$ are highlighted in bold.

Supplementary table 6. Summary of full-length spike binding IgG titer in COVID-19 patients stratified by age

Age	N[1]	Acute		Convalescent		12 months	
		Geomean (95% CI)	P value[2]	Geomean (95% CI)	P value	Geomean (95% CI)	P value
24-44[3]	6	260 (182-371)	Reference	12223 (6201-24093)	Reference	3955 (2596-6025)	Reference
24	2	184 (171-198)	0.5874	6182 (1900-20115)	0.9416	2656 (1693-4167)	0.8727
27	4	259 (158-424)	>0.9999	8317 (4082-16948)	0.9932	3481 (2031-5964)	0.9996
29	5	278 (185-417)	0.9997	11223 (5015-25115)	0.9998	3486 (2297-5290)	0.9995
35	5	281 (189-417)	0.9997	12419 (5414-28489)	>0.9999	4484 (2951-6813)	0.9995
41	5	278 (186-417)	0.9997	12719 (5744-28165)	>0.9999	3514 (1685-7330)	0.9997
44	5	232 (180-298)	0.9993	9954 (4347-22792)	0.9995	3032 (1602-5740)	0.9991
45	5	215 (163-283)	0.9916	19142 (4922-74443)	0.9993	5043 (1607-15824)	0.9996
47	5	160 (116-221)	0.5039	16082 (4302-60120)	0.9996	6257 (1970-19876)	0.9963
51	7	174 (129-235)	0.6699	9085 (3168-26058)	0.9994	3593 (1150-11223)	0.9998
52	9	239 (136-420)	0.9997	22851 (7060-73960)	0.9899	8958 (3147-25498)	0.8322
53	9	232 (131-409)	0.9996	31435 (9921-99606)	0.8532	12623 (4213-37820)	0.5339
61	10	266 (149-477)	>0.9999	32276 (11698-89052)	0.7546	13130 (4285-40236)	0.5173
62	10	294 (173-499)	0.9995	36697 (13410-100423)	0.6150	13787 (4507-42177)	0.4722
66	8	299 (151-590)	0.9996	73255 (37063-144785)	**0.0395**	27026 (15030-48598)	**0.0043**
68	6	219 (119-403)	0.9994	65139 (36033-117757)	**0.0491**	25238 (14020-45432)	**0.0127**
70	6	210 (112-395)	0.9993	52899 (28248-99064)	0.1079	19292 (12195-30520)	**0.0096**
72	7	312 (97-999)	0.9996	44258 (17645-111009)	0.3762	15028 (13242-17054)	**0.0100**
73	7	325 (102-1037)	0.9996	46958 (18406-119802)	0.3421	15682 (13436-18303)	**0.0077**
75	7	424 (138-1301)	0.9919	63985 (25669-159496)	0.1518	16173 (13587-19251)	**0.0064**

76	7	387 (119-1261)	0.9991	47522 (17980-125604)	0.3594	15629 (12699-19236)	**0.0072**
78	6	468 (124-1763)	0.9911	52064 (16813-161230)	0.4193	15970 (11980-21289)	**0.0108**
84	2	255 (33-1974)	>0.9999	46004 (4832-437952)	0.8983	n.a.[4]	n.a.

[1] N indicates the number of individuals included in the corresponding mini-moving-group. Individuals of the nearest 5 ages were grouped together as mini-moving-groups. Exceptions are age group 24 contains individuals aged 24 and 27; age group 27 contains individuals aged 24, 27, 29 and 35; age group 78 contains individuals aged 75, 76, 78 and 84; age group 84 contains individuals aged 78 and 84.

[2] IgG titers were Ln transformed in statistical analyses. Mixed-effects model and Dunnett's multiple comparisons were performed between all the mini-moving-groups and reference group. Adjusted P values are reported here and those P<0.05 are highlighted in bold.

[3] The group consisting of 6 individuals aged 24 to 44 was used as reference in Dunnett's multiple comparisons tests.

[4] Five subjects, including both individuals in age group 84, were excluded due to vaccination or sample missing at 12 months.

Supplementary table 7. Summary of S1 binding IgG titer in COVID-19 patients stratified by age

Age	N[1]	Acute		Convalescent		12 months	
		Geomean (95% CI)	P value[2]	Geomean (95% CI)	P value	Geomean (95% CI)	P value
24-44[3]	6	144 (124-166)	Reference	6494 (3101-13597)	Reference	2453 (1577-3816)	Reference
24	2	125 (88-178)	0.9861	3629 (1631-8078)	0.9413	1563 (721-3390)	0.9403
27	4	141 (113-175)	0.9999	4076 (2005-8288)	0.9895	2029 (1130-3643)	0.9994
29	5	144 (121-172)	>0.9999	5625 (2436-12987)	0.9997	2227 (1366-3629)	0.9996
35	5	153 (140-167)	0.9990	6723 (2730-16557)	>0.9999	2906 (2032-4154)	0.9992
41	5	143 (121-169)	>0.9999	7207 (3133-16579)	0.9998	2327 (1224-4425)	0.9999
44	5	140 (122-162)	0.9997	5623 (2325-13599)	0.9997	2016 (1143-3556)	0.9993
45	5	124 (94-164)	0.9850	9506 (3771-23966)	0.9991	2921 (1273-6703)	0.9996
47	5	129 (94-178)	0.9993	9610 (3778-24442)	0.9991	3578 (1395-9176)	0.9965
51	7	118 (92-153)	0.8902	4465 (1783-11181)	0.9991	1986 (677-5823)	0.9996
52	9	167 (94-297)	0.9993	10173 (3184-32500)	0.9991	4319 (1578-11822)	0.9780
53	9	156 (86-281)	0.9997	13240 (4243-41313)	0.9775	6113 (2042-18303)	0.7825
61	10	167 (99-280)	0.9993	14280 (5104-39954)	0.9286	6553 (2132-20142)	0.7351
62	10	161 (96-270)	0.9995	14616 (5198-41098)	0.9165	6521 (2123-20029)	0.7392
66	8	175 (93-331)	0.9992	31660 (16136-62121)	0.0991	13235 (7443-23536)	**0.0109**
68	6	125 (95-165)	0.9908	29904 (18800-47567)	0.0788	12401 (6828-22521)	**0.0295**
70	6	126 (96-165)	0.9916	29034 (18210-46294)	0.0865	9958 (7063-14038)	**0.0101**
72	7	118 (93-148)	0.8150	26773 (12692-56477)	0.2036	8679 (7411-10164)	**0.0155**
73	7	114 (91-143)	0.6772	26608 (12613-56129)	0.2071	8564 (7290-10060)	**0.0163**
75	7	129 (96-173)	0.9991	34736 (16147-74722)	0.1034	8464 (6963-10288)	**0.0163**

76	7	130<br>(97-175)	0.9992	29683<br>(14490-60809)	0.1407	9141<br>(7789-10727)	**0.0130**
78	6	138<br>(100-190)	0.9997	30409<br>(13040-70911)	0.1985	8519<br>(7600-9549)	**0.0189**
84	2	187<br>(120-292)	0.8985	47302<br>(15250-146715)	0.3744	n.a.[4]	n.a.

[1] N indicates the number of individuals included in the corresponding mini-moving-group. Individuals of the nearest 5 ages were grouped together as mini-moving-groups. Exceptions are age group 24 contains individuals aged 24 and 27; age group 27 contains individuals aged 24, 27, 29 and 35; age group 78 contains individuals aged 75, 76, 78 and 84; age group 84 contains individuals aged 78 and 84.

[2] IgG titers were Ln transformed in statistical analyses. Mixed-effects model and Dunnett's multiple comparisons were performed between all the mini-moving-groups and reference group. Adjusted P values are reported here and those P<0.05 are highlighted in bold.

[3] The group consisting of 6 individuals aged 24 to 44 was used as reference in Dunnett's multiple comparisons tests.

[4] Five subjects, including both individuals in age group 84, were excluded due to vaccination or sample missing at 12 months.

Supplementary table 8. Summary of S2 binding IgG titer in COVID-19 patients stratified by age

Age	N[1]	Acute		Convalescent		12 months	
		Geomean (95% CI)	P value[2]	Geomean (95% CI)	P value	Geomean (95% CI)	P value
24-44[3]	6	134 (106-169)	Reference	1526 (736-3167)	Reference	876 (633-1212)	Reference
24	2	123 (82-184)	0.9997	724 (306-1711)	0.8553	706 (522-954)	0.9687
27	4	122 (99-151)	0.9993	962 (576-1607)	0.9729	838 (612-1147)	0.9997
29	5	137 (104-181)	0.9999	1439 (595-3479)	0.9999	780 (588-1033)	0.9993
35	5	131 (99-173)	0.9999	1623 (672-3920)	0.9999	943 (661-1346)	0.9996
41	5	126 (91-174)	0.9996	1546 (597-3998)	>0.9999	756 (410-1393)	0.9995
44	5	128 (92-179)	0.9997	1450 (558-3769)	>0.9999	671 (387-1166)	0.9919
45	5	173 (96-312)	0.9923	1915 (699-5250)	0.9996	862 (396-1879)	>0.9999
47	5	160 (90-286)	0.9993	1635 (704-3797)	0.9999	1176 (496-2786)	0.9971
51	7	167 (96-290)	0.9990	1416 (714-2807)	0.9998	778 (339-1786)	0.9997
52	9	221 (101-486)	0.9267	3264 (1346-7917)	0.8956	1676 (754-3724)	0.8044
53	9	209 (94-468)	0.9698	4018 (1692-9539)	0.6797	2191 (969-4952)	0.4722
61	10	198 (94-419)	0.9832	4809 (2078-11128)	0.4602	2327 (1013-5349)	0.4221
62	10	197 (93-416)	0.9865	4903 (2125-11311)	0.4382	2275 (991-5223)	0.4457
66	8	198 (81-484)	0.9917	7309 (3343-15980)	0.1386	3978 (2699-5865)	**0.0015**
68	6	143 (81-251)	0.9997	6665 (3318-13386)	0.1544	4317 (2888-6452)	**0.0033**
70	6	153 (89-264)	0.9994	4683 (1823-12032)	0.5818	3074 (1667-5666)	0.0946
72	7	249 (100-616)	0.8820	4677 (1896-11538)	0.5483	2606 (1587-4281)	0.0610
73	7	246 (99-612)	0.8951	4898 (1987-12070)	0.5015	2369 (1371-4095)	0.1340
75	7	287 (120-686)	0.7013	6487 (2652-15865)	0.2668	2204 (1154-4212)	0.3128

76	7	291 (123-689)	0.6709	4790 (1916-11976)	0.5347	1639 (1132-2374)	0.2974
78	6	326 (122-874)	0.6616	6306 (2624-15156)	0.2836	1859 (1257-2749)	0.2299
84	2	192 (71-518)	0.9836	5026 (629-40142)	0.9117	n.a.[4]	n.a.

[1] N indicates the number of individuals included in the corresponding mini-moving-group. Individuals of the nearest 5 ages were grouped together as mini-moving-groups. Exceptions are age group 24 contains individuals aged 24 and 27; age group 27 contains individuals aged 24, 27, 29 and 35; age group 78 contains individuals aged 75, 76, 78 and 84; age group 84 contains individuals aged 78 and 84.

[2] IgG titers were Ln transformed in statistical analyses. Mixed-effects model and Dunnett's multiple comparisons were performed between all the mini-moving-groups and reference group. Adjusted P values are reported here and those P<0.05 are highlighted in bold.

[3] The group consisting of 6 individuals aged 24 to 44 was used as reference in Dunnett's multiple comparisons tests.

[4] Five subjects, including both individuals in age group 84, were excluded due to vaccination or sample missing at 12 months.

Supplementary table 9. Summary of neutralizing antibody titer in COVID-19 patients stratified by age

Age	N[1]	Acute		Convalescent		12 months	
		Geomean (95% CI)	P value[2]	Geomean (95% CI)	P value	Geomean (95% CI)	P value
24-44[3]	6	12.8 (7.89-20.77)	Reference	330.09 (142.34-765.5)	Reference	87.82 (41.11-187.59)	Reference
24	2	20.98 (4.91-89.6)	0.9913	420.45 (407.9-433.38)	0.9976	82.04 (34.84-193.19)	0.9999
27	4	14.48 (7.01-29.93)	0.9997	212.91 (85.81-528.25)	0.9991	57.98 (28.79-116.76)	0.9928
29	5	13.45 (7.52-24.04)	0.9999	247.39 (115.36-530.54)	0.9993	65.04 (36.16-117)	0.9992
35	5	13.45 (7.52-24.04)	0.9999	315.47 (113.25-878.77)	>0.9999	97.15 (39.6-238.33)	0.9998
41	5	10 (10-10)	0.9662	282.07 (102.11-779.22)	0.9997	81.26 (32.57-202.75)	0.9999
44	5	12.29 (8.21-18.4)	0.9999	300.87 (110.46-819.5)	0.9999	73.1 (28.29-188.84)	0.9996
45	5	20.55 (7.65-55.21)	0.9910	748.91 (234.81-2388.59)	0.9468	159.79 (46.7-546.79)	0.9921
47	5	30.42 (11.06-83.69)	0.7731	944.64 (298.63-2988.09)	0.8126	203.73 (59.01-703.32)	0.9417
51	7	28.24 (12.77-62.44)	0.6862	540.28 (160.29-1821.13)	0.9991	140.28 (42.38-464.36)	0.9991
52	9	43.05 (18.45-100.41)	0.2664	859.47 (287.38-2570.39)	0.8674	230.48 (87.54-606.84)	0.7718
53	9	38.39 (15.57-94.69)	0.4236	1079.17 (369.69-3150.21)	0.6613	334.48 (138-810.67)	0.3518
61	10	32.98 (14.27-76.24)	0.5251	1062.03 (393.5-2866.29)	0.6246	308.8 (133.75-712.98)	0.3848
62	10	27.11 (11.59-63.39)	0.7877	1160.36 (418.73-3215.51)	0.5569	298.85 (129.44-689.97)	0.4146
66	8	28.11 (10.06-78.57)	0.8619	1799.63 (784.83-4126.57)	0.1531	478.22 (269.85-847.48)	0.0626
68	6	14.91 (6.81-32.64)	0.9996	1839.42 (831.55-4068.86)	0.1437	545.77 (363.4-819.65)	**0.0359**
70	6	22.75 (8.21-63.05)	0.9736	1654.65 (738.4-3707.85)	0.1920	435.6 (288.12-658.57)	0.0684
72	7	33.57 (10.41-108.23)	0.7882	1538.29 (845.56-2798.54)	0.1470	394.99 (266.84-584.67)	0.0872
73	7	54.23 (15.87-185.32)	0.4287	1380.67 (806.11-2364.77)	0.1793	432.16 (301.02-620.43)	0.0661
75	7	74.69 (24.85-224.45)	0.1699	1797.67 (1052.49-3070.42)	0.0874	385.11 (272.52-544.23)	0.0919

76	7	74.69 (24.85-224.45)	0.1699	1345.25 (602.79-3002.2)	0.3020	368.38 (239.13-567.48)	0.1175
78	6	68.45 (18.91-247.72)	0.3401	1387.38 (538.09-3577.16)	0.3738	406.58 (235.41-702.21)	0.1265
84	2	30.66 (3.41-275.57)	0.9741	919.56 (53.28-15871.61)	0.9866	n.a.[4]	n.a.

[1] N indicates the number of individuals included in the corresponding mini-moving-group. Individuals of the nearest 5 ages were grouped together as mini-moving-groups. Exceptions are age group 24 contains individuals aged 24 and 27; age group 27 contains individuals aged 24, 27, 29 and 35; age group 78 contains individuals aged 75, 76, 78 and 84; age group 84 contains individuals aged 78 and 84.

[2] Neutralizing antibody titers were Ln transformed in statistical analyses. Mixed-effects model and Dunnett's multiple comparisons were performed between all the mini-moving-groups and reference group. Adjusted P values are reported here and those P<0.05 are highlighted in bold.

[3] The group consisting of 6 individuals aged 24 to 44 was used as reference in Dunnett's multiple comparisons tests.

[4] Five subjects, including both individuals in age group 84, were excluded due to vaccination or sample missing at 12 months.

Supplementary table 10. Summary of antibody responses in COVID-19 patients stratified by age

Age	N[1]	Full-length spike binding IgG	S1 domain binding IgG	S2 domain binding IgG	Neutralizing antibody
		Convalescent[2]	Convalescent	Convalescent	Convalescent
24	2	0.0013	0.0004	0.3711	0.0745
27	4	<0.0001	<0.0001	0.0026	0.0052
29	5	<0.0001	<0.0001	<0.0001	0.0002
35	5	<0.0001	<0.0001	<0.0001	<0.0001
41	5	<0.0001	<0.0001	<0.0001	<0.0001
44	5	<0.0001	<0.0001	<0.0001	<0.0001
45	5	<0.0001	<0.0001	<0.0001	<0.0001
47	5	<0.0001	<0.0001	<0.0001	<0.0001
51	7	<0.0001	<0.0001	<0.0001	<0.0001
52	9	<0.0001	<0.0001	<0.0001	<0.0001
53	9	<0.0001	<0.0001	<0.0001	<0.0001
61	10	<0.0001	<0.0001	<0.0001	<0.0001
62	10	<0.0001	<0.0001	<0.0001	<0.0001
66	8	<0.0001	<0.0001	<0.0001	<0.0001
68	6	<0.0001	<0.0001	<0.0001	<0.0001
70	6	<0.0001	<0.0001	<0.0001	<0.0001
72	7	<0.0001	<0.0001	<0.0001	<0.0001
73	7	<0.0001	<0.0001	<0.0001	<0.0001
75	7	<0.0001	<0.0001	<0.0001	<0.0001
76	7	<0.0001	<0.0001	<0.0001	<0.0001
78	6	<0.0001	<0.0001	<0.0001	<0.0001
84	2	<0.0001	<0.0001	0.0004	0.0208

[1] N indicates the number of individuals included in the corresponding mini-moving-group. Individuals of the nearest 5 ages were grouped together as mini-moving-groups. Exceptions are age group 24 contains individuals aged 24 and 27; age group 27 contains individuals aged 24, 27, 29 and 35; age group 78 contains individuals aged 75, 76, 78 and 84; age group 84 contains individuals aged 78 and 84.

[2] IgG titers and neutralizing antibody titers were Ln transformed in statistical analyses. RM two-way ANOVA and Bonferroni's multiple comparisons were performed between acute and convalescent phases for all the mini-moving-groups. Adjusted P values are reported here and those P<0.05 are highlighted in bold.

Supplementary table 11. The demographics of patients enrolled in the study

	All patients	Severity[3] 2	Severity 4	Severity 5	Severity 6	Severity 7
No. of subjects	29[4]	1	13	13	1	1
Sex, M/F	14/15	1/0	7/6	5/8	0/1	1/0
Median age, years (range)	61 (24-84)	41	53 (30-84)	62 (24-79)	22	53
Comorbidity[1] (%)	19 (66)	0 (0)	8 (62)	9 (69)	1 (100)	1 (100)
Immunosuppression[2] (%)	13 (45)	0 (0)	7 (54)	6 (46)	0 (0)	0 (0)

[1] Comorbidities include chronic heart disease, chronic lung disease, chronic liver disease, chronic kidney disease, diabetes, cancer, rheumatic disease, neurological disease, and autoimmune disease.

[2] Inherent immunosuppressive disease, HIV, organ transplant, chemotherapy, Prednisone or other immunosuppressive medication.

[3] COVID-19 disease severity score was modified as follows, severity 2: home-isolated with symptoms; severity 4: hospitalised with medical needs; severity 5: hospitalised needing oxygen; severity 6: hospitalised needing ventilation; severity 7: hospitalised needing respirator.

[4] Five subjects at 12 months were excluded due to vaccination or sample missing, n=24 at 12 months.
