# Supplementary material for: Impact of ageing on homologous and human-coronavirus-reactive antibodies after SARS-CoV-2 vaccination or infection
Source: NPJ Vaccines. 2024 Feb 20;9:37. doi: 10.1038/s41541-024-00817-z (PMC10879087; doi:10.1038/s41541-024-00817-z)
Supplement: Supplementary file 2 — Reporting Summary [file 41541_2024_817_MOESM2_ESM.pdf]

nature portfolio

Corresponding author(s): F. Zhou

Last updated by author(s): Jan 11, 2024

Reporting Summary

Nature Portfolio wishes to improve the reproducibility of the work that we publish. This form provides structure for consistency and transparency in reporting. For further information on Nature Portfolio policies, see our Editorial Policies and the Editorial Policy Checklist.

Statistics

For all statistical analyses, confirm that the following items are present in the figure legend, table legend, main text, or Methods section.

n/a	Confirmed	
☐	☒	The exact sample size (*n*) for each experimental group/condition, given as a discrete number and unit of measurement
☐	☒	A statement on whether measurements were taken from distinct samples or whether the same sample was measured repeatedly
☐	☒	The statistical test(s) used AND whether they are one- or two-sided<br>*Only common tests should be described solely by name; describe more complex techniques in the Methods section.*
☐	☒	A description of all covariates tested
☐	☒	A description of any assumptions or corrections, such as tests of normality and adjustment for multiple comparisons
☐	☒	A full description of the statistical parameters including central tendency (e.g. means) or other basic estimates (e.g. regression coefficient) AND variation (e.g. standard deviation) or associated estimates of uncertainty (e.g. confidence intervals)
☐	☒	For null hypothesis testing, the test statistic (e.g. *F*, *t*, *r*) with confidence intervals, effect sizes, degrees of freedom and *P* value noted<br>*Give P values as exact values whenever suitable.*
☒	☐	For Bayesian analysis, information on the choice of priors and Markov chain Monte Carlo settings
☒	☐	For hierarchical and complex designs, identification of the appropriate level for tests and full reporting of outcomes
☐	☒	Estimates of effect sizes (e.g. Cohen's *d*, Pearson's *r*), indicating how they were calculated

Our web collection on statistics for biologists contains articles on many of the points above.

Software and code

Policy information about availability of computer code

Data collection	Enzyme-linked immunosorbent assay and microneutralization assay data were collected using a synergy H1 plate reader, BioTek. Virus neutralization data were collected using an optical microscopy.
Data analysis	Microsoft Excel for Mac, version 16.77.1, and GraphPad Prism 9 for Mac, version 9.5.1.

For manuscripts utilizing custom algorithms or software that are central to the research but not yet described in published literature, software must be made available to editors and reviewers. We strongly encourage code deposition in a community repository (e.g. GitHub). See the Nature Portfolio guidelines for submitting code & software for further information.

Data

Policy information about availability of data

All manuscripts must include a data availability statement. This statement should provide the following information, where applicable:

- Accession codes, unique identifiers, or web links for publicly available datasets
- A description of any restrictions on data availability
- For clinical datasets or third party data, please ensure that the statement adheres to our policy

The data that support the finding of this study are available from the corresponding author upon reasonable request.

Research involving human participants, their data, or biological material

Policy information about studies with human participants or human data. See also policy information about sex, gender (identity/presentation), and sexual orientation and race, ethnicity and racism.

Reporting on sex and gender	The findings are not limited to only one sex. Biologic sex was not considered in the study design. Sex refers to biological sex assigned at birth and the information was collected using electronic case report forms from each and every study participant. All participants provided written informed consent before inclusion in the study. The study involved a total of 46 male participants and 63 female participants. The findings of the study were not associated with sex.
Reporting on race, ethnicity, or other socially relevant groupings	Race, ethnicity or other socially relevant groupings were not considered in the study design. Confounding variables regarding race were not controlled for due to the small sample size.
Population characteristics	The study population consists of 109 participants, 46 male and 63 female, age range from 24 to 98 years old. Sixty-seven participants reported any comorbidity, including chronic heart disease, chronic lung disease, chronic liver disease, chronic kidney disease, diabetes, cancer, rheumatic disease, neurological disease, and autoimmune disease.
Recruitment	The vaccinees were recruited before receiving pandemic COVID-19 mRNA vaccine. No vaccinees had tested rever transcription polymerase chain reaction (rt-PCR) positive for SARS-CoV-2 or had any COVID-19 symptom before receiving the first dose vaccine.<br>The COVID-19 patients were recruited after tested rt-PCR positive for SARS-CoV-2 from nasopharyngeal swabs during March and April 2020. None of the infected patients received any COVID-19 vaccine within 12 months post diagnosis.
Ethics oversight	Regional Committee for Medical Research Ethics, Western Norway (REK Vest number 118664) and Northern Norway (REK Nord number 218629).

Note that full information on the approval of the study protocol must also be provided in the manuscript.

Field-specific reporting

Please select the one below that is the best fit for your research. If you are not sure, read the appropriate sections before making your selection.

☒ Life sciences ☐ Behavioural & social sciences ☐ Ecological, evolutionary & environmental sciences

For a reference copy of the document with all sections, see nature.com/documents/nr-reporting-summary-flat.pdf

Life sciences study design

All studies must disclose on these points even when the disclosure is negative.

Sample size	Sample size was determined based on the number of participants recruited and met the study criteria.
Data exclusions	Five COVID-19 patients were excluded at 12 months due to vaccination or sample missing.
Replication	Biological replicates were used in all experiments.
Randomization	Samples were allocated into groups of vaccinees or patients based on the COVID-19 vaccination and infection history collected in the collected using electronic case report forms.
Blinding	The investigator performing serological experiments were blinded to the COVID-19 vaccination and infection history of the study participants.

Reporting for specific materials, systems and methods

We require information from authors about some types of materials, experimental systems and methods used in many studies. Here, indicate whether each material, system or method listed is relevant to your study. If you are not sure if a list item applies to your research, read the appropriate section before selecting a response.

Materials & experimental systems

n/a	Involved in the study
☐	☒ Antibodies
☐	☒ Eukaryotic cell lines
☒	☐ Palaeontology and archaeology
☒	☐ Animals and other organisms
☐	☒ Clinical data
☒	☐ Dual use research of concern
☒	☐ Plants

Methods

n/a	Involved in the study
☒	☐ ChIP-seq
☒	☐ Flow cytometry
☒	☐ MRI-based neuroimaging

Antibodies

Antibodies used: Anti-human IgG, Sigma-Aldrich, Cat: I-3382; Biotin labelled anti-human IgG, Sigma-Aldrich, Cat: B-1140; Rabbit monoclonal IgG against SARS-CoV-2 NP, Sino Biological, Cat: 40143-R019; Biotinylated goat anti-rabbit IgG (H+L), Southern Biotech, Cat: 4050-08.

Validation: IgG from human serum, Sigma-Aldrich, Cat: I-4506 was used as positive control for the anti-human IgG and biotin labelled anti-human IgG in enzyme-linked immunosorbent assay. Vero cells infected with SARS-CoV-2 was used as positive control for rabbit monoclonal IgG against SARS-CoV-2 NP and biotinylated goat anti-rabbit IgG (H+L) in microneutralization assay.

Eukaryotic cell lines

Policy information about cell lines and Sex and Gender in Research

Cell line source(s): Vero cells were obtained from ATCC, Cat: CCL-81. LLC-MK2 cells were obtained from ATCC, Cat: CCL-7. HCT-8 cells were obtained from ATCC, Cat: CCL-244. Expi293F cells were obtained from Thermo Fisher Scientific, Cat: A14527.

Authentication: All cell lines were obtained commercially and were not authenticated.

Mycoplasma contamination: All cells were tested negative for mycoplasma.

Commonly misidentified lines (See ICLAC register): No commonly misidentified cell lines were used.

Clinical data

Policy information about clinical studies
All manuscripts should comply with the ICMJE guidelines for publication of clinical research and a completed CONSORT checklist must be included with all submissions.

Clinical trial registration: The study is registered in the National Institute for Health database Clinical trials.gov (NCT04706390).

Study protocol: The clinical trial study protocol is available from the corresponding author upon reasonable request.

Data collection: Electronic case report forms were used to collect demographics, comorbidities, infection history (re-PCR test results and presence of COVID-19 symptoms), vaccination data and side reactions. The vaccinees were recruited January 2021. The patients were recruited March to April 2020.

Outcomes: Study outcomes were the impact of ageing on SARS-CoV-2 specific and HCoV cross-reactive antibody responses in COVID-19 vaccinees and patients.

Plants

Seed stocks: n/a

Novel plant genotypes: n/a

Authentication: n/a
